# Fabrication of RIG-I-Activating Nanoparticles for Intratumoral Immunotherapy via Flash Nanoprecipitation

**DOI:** 10.1021/acs.molpharmaceut.5c00125

**Published:** 2025-07-01

**Authors:** Payton T. Stone, Alexander J. Kwiatkowski, Eric W. Roth, Olga Fedorova, Anna M. Pyle, John T. Wilson

**Affiliations:** † Department of Chemical and Biomolecular Engineering, 5718 Vanderbilt University, Nashville, Tennessee 37235, United States; ‡ Department of Biomedical Engineering, 5718 Vanderbilt University, Nashville, Tennessee 37235, United States; § NUANCE BioCryo, 3270 Northwestern University, Evanston, Illinois 60208, United States; ∥ Department of Molecular, Cellular, and Developmental Biology, 5755 Yale University, New Haven, Connecticut 06520, United States; ⊥ 607904 Howard Hughes Medical Institute, Chevy Chase, Maryland 20815, United States; # Department of Chemistry, 5755 Yale University, New Haven, Connecticut 06520, United States; ∇ Vanderbilt Center for Immunobiology, Vanderbilt University Medical Center, Nashville, Tennessee 37232, United States; ○ Vanderbilt Institute for Infection, Immunology, and Inflammation, Vanderbilt University Medical Center, Nashville, Tennessee 37232, United States; ◆ Vanderbilt Institute of Chemical Biology, 5718 Vanderbilt University, Nashville, Tennessee 37232, United States; ¶ Vanderbilt Institute of Nanoscale Science and Engineering, 5718 Vanderbilt University, Nashville, Tennessee 37232, United States; & Vanderbilt-Ingram Cancer Center, Vanderbilt University Medical Center, Nashville, Tennessee 37232, United States

**Keywords:** nanoparticle, flash nanoprecipitation, cancer immunotherapy, RIG-I, drug delivery, polymer

## Abstract

Intratumoral immunotherapy is a promising strategy for stimulating local and systemic antitumor immunity while eliminating or reducing immune-related adverse events often attendant to systemic administration. Activation of the cytosolic pattern recognition receptor retinoic acid-inducible gene I (RIG-I) at tumor sites stimulates innate immunity that can potentiate a T cell-dependent adaptive antitumor immune response. However, the activity and efficacy of 5′-triphosphate RNA (3pRNA) agonists of RIG-I are hindered by poor in vivo stability, rapid degradation, limited cellular uptake, and inefficient cytosolic delivery. To overcome these challenges, we developed RIG-I-activating nanoparticles (RANs) assembled using a flash nanoprecipitation (FNP) process to load a potent stem-loop 3pRNA (SLR) RIG-I agonist into endosome-destabilizing polymeric nanoparticles. We leveraged FNP to induce turbulent micromixing among a corona-forming poly­(ethylene glycol)-*block*-(dimethylaminoethyl methacrylate-*co*-butyl methacrylate) (PEG-DB) diblock copolymer, a hydrophobic core-forming DB counterpart, and an SLR RIG-I agonist, resulting in the self-assembly of densely loaded nanoparticles that promoted endosomal escape and cytosolic delivery of 3pRNA cargo. Through optimization of polymer properties and inlet feed ratios, we developed RANs with high and improved loading efficiency and increased serum stability relative to a previously reported micelleplex formulation assembled via electrostatic complexation with PEG-DB polymers. We found that optimized RANs exhibited potent immunostimulatory activity in vitro and in vivo when delivered intratumorally. As a result, in preclinical models of MC38 colon cancer and B16.F10 melanoma, intratumoral administration of RANs suppressed tumor growth and increased survival time relative to vehicle controls. Collectively, this work demonstrates that FNP can be harnessed as a versatile and scalable process for the efficient loading of nucleic acids into polymeric nanoparticles and highlights the potential of RANs as a translationally promising platform for intralesional cancer immunotherapy.

## Graphical abstract

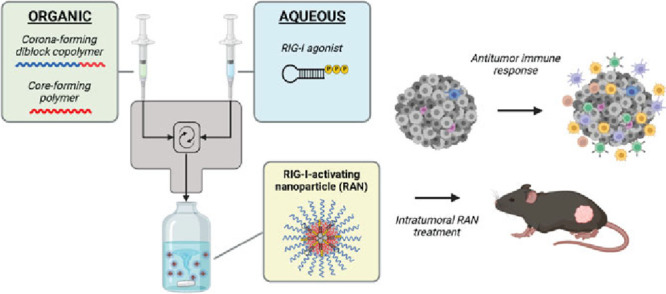

## 1 Introduction

Intratumoral immunotherapy is a promising strategy for stimulating adaptive immunity against tumor antigens and can offer advantages over systemic administration, including a wider therapeutic window due to localized delivery directly to the tumor site, in turn mitigating the risks of off target-toxicity, systemic inflammation, and other immune-related adverse events (irAEs).^1–5^ For tumors that are readily accessible, intratumoral administration can be routinely implemented into treatment regimens, as exemplified by oncolytic virus therapy for melanoma (T-VEC).^6^ While direct injection into tumors can be challenging for some cancer types or metastatic sites, advances in interventional radiology and implantable drug delivery devices are opening new opportunities for local and/or intralesional therapy for an increasing number of patients.^7,8^ Among the most promising and widely investigated class of intratumoral agents are nucleic acid agonists of pattern recognition receptors (PRRs) which trigger acute inflammatory responses that potentiate local and systemic antitumor immunity via multiple mechanisms. For example, CpG DNA, a TLR-9 agonist, has been explored extensively for intratumoral administration with ongoing clinical trials of vidutolimod demonstrating promising results in treating advanced melanoma cases.^9,10^ Another promising but less explored PRR target is retinoic acid-inducible gene I (RIG-I), a viral sensor that recognizes and binds to 5′-triphosphorylated RNA (3pRNA) present within the cytosol. Activation of RIG-I elicits a conformational change and release of signaling domains (CARDs) which can interact with an adapter protein (MAVS), inducing the secretion of type-I interferons (IFN-I) and other pro-inflammatory mediators which can stimulate antitumor innate immunity while kick-starting adaptive immune responses by priming antigen-specific cytotoxic T lymphocytes.^11–14^ Additionally, RIG-I agonists can induce apoptosis in some cancer cells, directly contributing to tumor eradication and stimulating the release of neoantigens which can further amplify antigen-specific T cell responses.^15,16^ Furthermore, RIG-I pathway activation has been found to directly correlate with response to anti-CTLA-4 immune checkpoint blockade (ICB) and prolonged survival in some cancers.^1,17–20^ This has motivated the investigation of RIG-I agonists for cancer immunotherapies with promising preclinical results in multiple tumor models, including melanoma, breast, and colon cancers, to name a few.^17,21,22^

Despite such promise as an immunostimulatory agent, free 3pRNA faces major delivery challenges in vivo that limit its clinical efficacy including susceptibility to nuclease degradation, rapid clearance from the injection site, and, critically, an inability to permeate the cell or endosomal membranes to enter the cytosol, where RIG-I is located.^23–26^ To address these barriers in phase I clinical trials, 3pRNA (MK-4621) was electrostatically complexed with jetPEI, a polyethylenimine transfection agent, with evidence that MK-4621 activated the RIG-I pathway. However, no clinical benefit was observed at the doses tested.^27,28^ While poor responses may be attributed to several factors, this has begun to motivate efforts focused on designing next-generation delivery systems capable of improving the activity and delivery of 3pRNA, including platforms for promoting more efficient cytosolic delivery and protecting 3pRNA from premature degradation in the extracellular environment. Notable examples include the work of Huang and co-workers who described 3pRNA-loaded lipid calcium phosphate nanoparticles for pancreatic cancer immunotherapy^29^ and Hou et al., who complexed 3pRNA to cationic aluminum hydroxide as a vaccine adjuvant, an application also pursued by Koerner et al., Toy et al., and Levy et al., who leveraged PLGA-based micro- and nanoparticle formulations for 3pRNA delivery.^30–33^ Additionally, our group has recently repurposed lipid nanoparticles (LNPs) for improving 3pRNA delivery while demonstrating efficacy in multiple tumor models.^34^

Though promising, these strategies have largely leveraged existing materials, while research centered on developing carriers optimized for 3pRNA delivery has been considerably more limited, particularly in relation to other classes of RNA therapeutics.^35,36^ Toward filling this gap, our group has recently described a library of pH-responsive polymers for 3pRNA delivery based on poly­[(ethylene glycol monomethyl ether) (mPEG)-*b*-(dimethylaminoethyl methacrylate (DMAEMA)-*co*-alkyl methacrylate)] (mPEG-*b*-DA) diblock copolymers comprising protonatable DMAEMA groups and pendant alkyl chains with lengths between 2 and 12 carbons and backbone densities between 0 and 60 mol %.^35^ The cationic DMAEMA groups in the hydrophobic second block are used to electrostatically complex 3pRNA, resulting in the formation of PEGylated “micelleplexes” that can facilitate endosomal escape of 3pRNA to the cytosol, culminating in RIG-I activation. While this represents among the first studies to pursue the rational design of carriers for RIG-I agonists, the platform was limited by the relatively high amount of polymer required to adequately complex the 3pRNA cargo, as well as suboptimal serum stability. Additionally, the capacity of this family of 3pRNA carriers to activate RIG-I following intratumoral administration or to confer therapeutic benefit in tumor models has not yet been explored.

Herein, we build upon our previous work to further optimize and advance next-generation RIG-I-activating nanoparticles (RANs) and evaluate this platform for intratumoral immunotherapy. We selected one of the lead endosomolytic polymers that emerged from our previous library screen—DMAEMA_50%_-*co*-butyl methacrylate_50%_ (referred to henceforth as DB)—and leveraged flash nanoprecipitation (FNP) via confined impingement jet (CIJ) mixing to form core–shell nanoparticles loaded with 3pRNA (Figure 1). FNP induces turbulent micromixing between a polymer-containing organic stream and a hydrophilic drug-containing aqueous stream resulting in the nucleation, precipitation, and self-assembly of drug-loaded nanoparticles with uniform size and morphologies.^36–39^ This process has been employed for assembling polymeric nanoparticles of various morphologies such as micelles, fibromicelles, polymersomes, multicompartmental vesicles (MCVs), and bicontinuous nanospheres (BCNs), and opens an expansive parameter space for immunotherapy applications.^36,38,40^ Indeed, a similar CIJ mixer has been used for large-scale production of mRNA-loaded lipid nanoparticles as COVID-19 vaccines.^41^ With this in mind, we devised a RAN formulation method by which an organic solution comprising a corona-forming PEG-*b*-DB diblock polymer and core-forming DB chains were impinged against an aqueous stream containing 3pRNA cargo, here SLR14, a potent and selective 14-base-pair stem-loop 3pRNA (SLR) RIG-I agonist.^11,17^ We demonstrated that this scalable process yields potently immunostimulatory RANs, capable of delivering SLR14 to innate immune cells in the TME upon intratumoral administration, resulting in RIG-I activation that confers therapeutic efficacy in multiple tumor models. In doing so, we have highlighted the ability of FNP to serve as a scalable, tunable, and efficient fabrication process for the polymeric delivery of novel RIG-I agonists for intralesional immunotherapies. This work underscores the potential for FNP to advance the development of versatile drug delivery platforms, paving the way for more effective cancer treatments while working to address clinical shortcomings in cancer immunotherapies.

**Figure 1:**
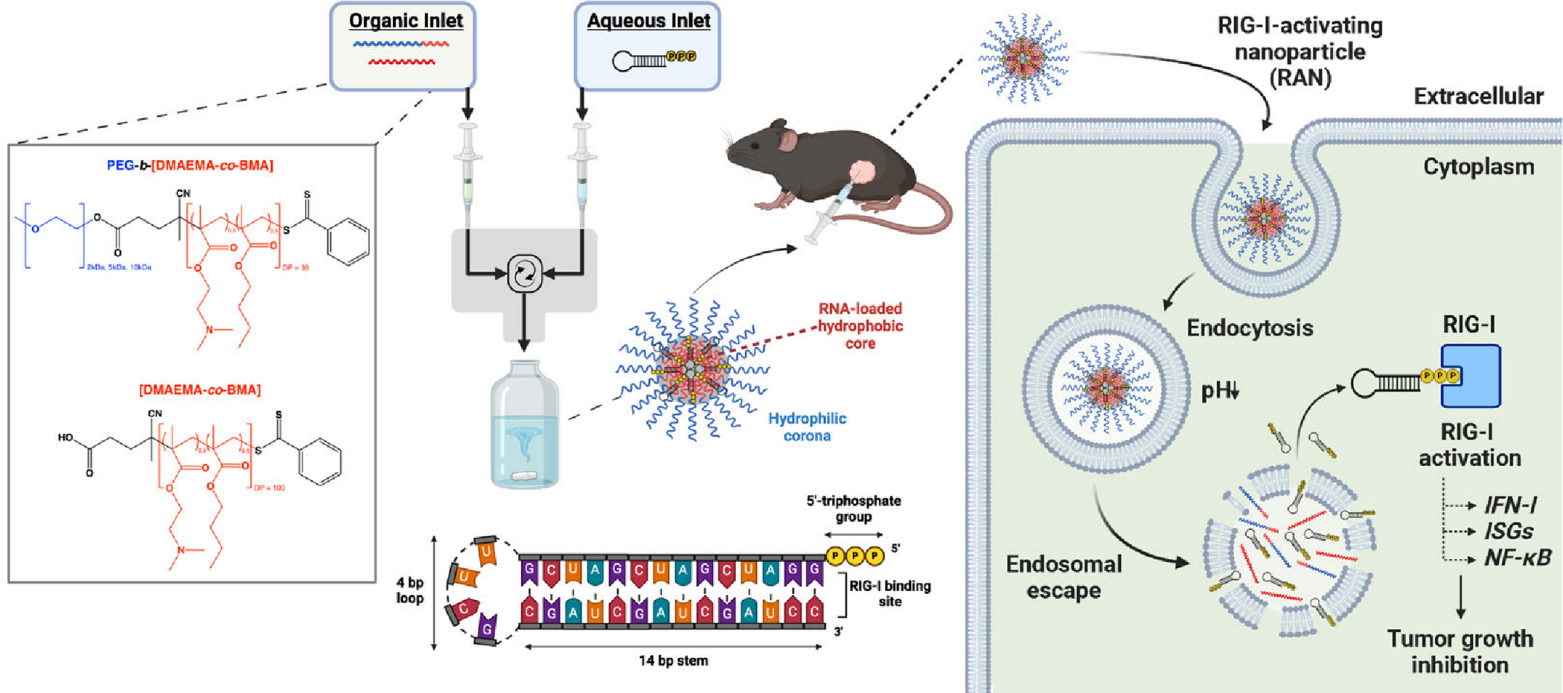
Design, synthesis, and fabrication of RIG-I-activating nanoparticles (RANs). RAFT polymerization was used to synthesize pH-responsive, endosomolytic mPEG-*block*-[DMAEMA_50%_-*co*-BMA_50%_]_6 kDa_ (PEG-DB) corona-forming diblock copolymers with mPEG molecular weights of 2, 5, and 10 kDa and a [DMAEMA-*co*-BMA]_15 kDa_ (DB) core-forming copolymer (15 kDa) (created with ChemDraw 20.1.0.112). Flash nanoprecipitation (FNP) was employed to induce turbulent micromixing within a confined impingement jet (CIJ) mixer, facilitating particle self-assembly and encapsulation of 5′-triphosphorylated RNA (3pRNA) cargos within nanoparticles, resulting in the formation of RANs. After purification, RANs were administered intratumorally to stimulate an innate immune response that inhibited tumor growth and prolonged survival in murine cancer models. Upon administration, cells within the tumor microenvironment (TME) endocytose RANs, which disassemble and expose membrane lytic DB segments in response to a decrease in pH within the endosome. This culminates in endosomal disruption and subsequent release of triphosphorylated RNA into the cytosol where it can bind to and activate RIG-I to elicit antitumor innate immunity (created with Biorender.com).

## 2 Experimental Section

### 2.1 Materials

All materials were purchased from Sigma-Aldrich unless otherwise specified.

### 2.2 Methods

#### 2.2.1 Polymer Synthesis

The reversible addition–fragmentation chain transfer (RAFT) polymerization used in this study is shown in Figure 1 and has been described previously.^69^ Briefly, for the surface-forming polymers, butyl methacrylate (BMA) and 2-(dimethylamino) ethyl methacrylate (DMAEMA) (Tokyo Chemical Industry) monomers were filtered and reacted with poly­(ethylene glycol) 4-cyano-4-(phenylcarbonothioylthio)­pentanoate (PEG-CTA) in the presence of 4-4′-AZO-bis­(4-cyanovaleric acid) initiator (V501) at 70 °C for 18 h in dioxane after a 30 min purge under nitrogen gas. For core-forming polymers, butyl methacrylate (BMA) and 2-(dimethylamino) ethyl methacrylate (DMAEMA) (Tokyo Chemical Industry) monomers were filtered and reacted with 4-cyano-4-(phenylcarbonothioylthio)­pentanoate (CTA) in the presence of 4-4′-AZO-bis­(4-cyanovaleric acid) initiator (V501) at 70 °C for 18 h in dioxane after a 30 min purge under nitrogen gas. The CTA:initiator ratio was 5:1 and the monomer weight fraction was 0.2 for all polymerizations. Polymerizations were terminated by supplementing the reaction mixtures with oxygen, and polymers were purified via dialysis through an acetone–water gradient in 3.5 kDa MWCO SnakeSkin dialysis tubing (Thermo Fisher Scientific). Specifically, polymers were purified in 100% acetone twice, 1:1 acetone:water once, and 100% water twice over 48 h. Polymers were then lyophilized for 48 h. ^1^H NMR spectroscopy in CDCl_3_ was conducted on the post-polymerization reaction mixture to determine conversion and on the final lyophilized product to determine composition using a 400 MHz NMR Spectrometer (Bruker), courtesy of the Vanderbilt University Small Molecule NMR Facility Core. Polymer properties are included in Table 1.

**Table 1:** Polymer Library and Characterization

polymer	target DP	actual DP^a^	polymer *M*_W_ ^b^ (kDa)	%DMAEMA^c^ (mol %)	%BMA^c^ (mol %)	polymer type
PEG_2 kDa_-DB	35	35	7.2	53.3	46.7	corona-forming
PEG_5 kDa_-DB	35	45	11.7	54.9	45.1	corona-forming
PEG_10 kDa_-DB	35	36	15.4	53.8	46.2	corona-forming
DB	100	92	13.8	54.5%	45.5%	core-forming

a Degree of polymerization (DP) as calculated by conversion ^1^H NMR.

b Number-average molecular weight (*M*_n_) as calculated by conversion ^1^H NMR.

c Composition as determined by ^1^H NMR analysis of purified polymer.

#### 2.2.2 Nanoparticle Formulation Using Flash Nanoprecipitation

A confined impingement jet (CIJ) mixer (Holland Applied Technologies) was used to fabricate drug-loaded nanoparticles. Briefly, surface-forming and core-forming polymers were dissolved in acetonitrile in a 1:1 ratio at 1 mg/mL. Stem-loop-RNAs (SLRs) were dissolved in ultrapure distilled RNase/DNase-free H_2_O (Invitrogen) at a 8:1 ratio of polymer nitrogen moles to SLR14 phosphate moles (N:P ratio). Both solutions were aspirated into 1 mL disposable polypropylene syringes (Fisher) and attached to the inlets of the CIJ. Turbulent micromixing was induced by rapidly impinging the contents of the syringes simultaneously into the CIJ. The resultant mixture was collected in a 20 mL scintillation vial (Fisher) containing 2× volume of ultrapure distilled RNase/DNase-free H_2_O under rapid stirring. Nanoparticles were then purified and concentrated for future experiments using 50 kDa MWCO 15 mL spin filters (Amicon). Nanoparticles were stored at 4 °C for up to 3 weeks. A Genesys UV–visible Spectrophotometer (Thermo Fisher Scientific) was used to measure sample absorbance at 298 nm, and polymer concentration was determined via linear interpolation of absorbance readings using a standard curve of known polymer concentrations. SLR14 concentrations of RAN formulations were determined using a Quant-it RiboGreen RNA assay kit (Thermo Fisher Scientific). Formulations were diluted in 1× PBS to desired concentrations prior to treatment.

#### 2.2.3 Dynamic Light Scattering

Size, PDI, and zeta potential measurements were obtained using a Zetasizer Ultra (Malvern Panalytical), courtesy of the Vanderbilt Institute of Nanoscale Science and Engineering (VINSE). Size and PDI measurements were obtained by diluting the nanoparticles 100× in sterile-filtered phosphate-buffered saline (PBS) (Gibco) into a 1.5 mL semimicro cuvette (Thermo Fisher Scientific). Zeta potential measurements were obtained by diluting the nanoparticles 100× in 10 mM NaCl solution into a DTS1070 capillary cell. ZS Explorer software was used for all measurements.

#### 2.2.4 Transmission Electron Microscopy

Transmission electron microscopy (TEM) was conducted using a Tecnai Osiris analytical 60–200 kV scanning/transmission electron microscope, courtesy of VINSE. Samples were drop-casted on 300 mesh copper grids (Ted Pella Inc.), stained with methylamine tungstate, and allowed to sit overnight. The following day, images were captured on the TEM at minimum contrast using a 200 kV FEG register and a 20 μM objective aperture. Images were exported and analyzed using TIA software.

#### 2.2.5 Cryogenic Electron Microscopy

Cryogenic electron microscopy imaging was conducted using a method described previously with minor adaptations for this study.^39^ Prior to plunge-freezing, 200 mesh Cu grids with a lacey carbon membrane (EMS Cat# LC200-CU-100) were glow-discharged in a Pelco easiGlow glow discharger (Ted Pella Inc.) using an atmosphere plasma generated at 15 mA for 15 s with a pressure of 0.24 mbar. This treatment created a negative charge on the carbon membrane, allowing aqueous samples to spread evenly over of the grid. 4 μL of sample was pipetted onto the grid and blotted for 5 s with a blotting pressure of 1, followed by immediate plunging into liquid ethane within an FEI Vitrobot Mark IV plunge freezing instrument (Thermo Fisher Scientific). Grids were then transferred to liquid nitrogen for storage. The plunge-frozen grids were kept vitreous at −180 °C in a Gatan ELSA cryo transfer holder (Gatan Inc.) while viewing in a JEOL JEM1400 LaB6 emission TEM (JEOL Inc.) at 120 keV. Image data was collected by a Gatan OneView camera (Gatan Inc.).

#### 2.2.6 Galectin8 Reporter Assays

Galectin8 (Gal8) reporter assays were conducted as previously described with minor modifications.^54^ Briefly, MDA-MB-231 human breast adenocarcinoma cells expressing a Gal8-YFP fusion protein were seeded at a density of 5000 cells per well in 96-well black-walled, clear-bottom TC-treated plates (Greiner) and allowed to incubate overnight. Once adhered, cells were treated with RAN nanoparticle formulations at 10× dosage (20 μL into 180 μL of cells) or PBS as a control. Polymer concentrations were determined via UV–vis spectrophotometry, and cells were treated dose-dependently using a 2-fold serial dilution with a highest dose of 50 μM. After 24 h, the media in each well was discarded and replaced with Fluorobrite DMEM imaging media (200 μL) (Gibco) supplemented with 1:5000 Hoechst nuclear stain (Thermo Fisher Scientific). Cells were imaged using an ImageXpress Nano Automated Imaging System with a 20× Nikon CFI60 series objective (Molecular Devices), courtesy of the Vanderbilt High Throughput Screening Facility. Images were then analyzed using MetaXpress software (Molecular Devices) which quantified the number and intensity of Gal8-YFP pixels, representing points of endosome disruption. A nonlinear regression curve was fit to the data points, and EC_50_ values were obtained using GraphPad Prism10.2.3 software.

#### 2.2.7 In Vitro Evaluation of Immunostimulatory Activity in Reporter Cells

A549-Dual reporter cells were seeded in 96-well, clear-bottom TC-treated plates at a density of 5000 cells per well and allowed to adhere overnight. The next day, RNA concentrations of RAN formulations were determined using a Quant-it RiboGreen RNA assay kit (Thermo Fisher Scientific), cells were treated in a dose-dependent manner via a 2-fold serial dilution with nanoparticle formulations at 10x desired dosage (20 μL into 180 μL of cells), and after 24 h interferon expression was measured using a Synergy HI plate reader (Bio-Tek) and a QUANTI-Luc luciferase-based detection assay (InvivoGen). Luminescent readouts for treated groups were normalized to PBS control wells and reported as relative luminescence (RLUs). A nonlinear regression curve was fit to the data points, and EC_50_ values were obtained using GraphPad Prism10.2.3 software.

#### 2.2.8 In Vitro Evaluation of BMDM Activation

Bone marrow-derived macrophages (BMDMs) were harvested from six-week-old C57/BL6 mice (Jackson Laboratory) and allowed to culture in 100 mm TC-treated Petri dishes (Corning) supplemented with 20 ng/mL of GM-CSF. On Day 3, an additional 5 mL of fresh media supplemented with 20 ng/mL M-CSF was added. On Day 5, 10 mL of cell culture suspension per dish was collected, centrifuged at 1500 rpm, resuspended in fresh media supplemented with 20 ng/mL M-CSF, and returned to each dish. On Day 7, cells were lifted from the dishes, seeded in 96-well TC-treated plates at a density of 100,000 cells per well, and allowed to adhere overnight. The next day, RNA concentrations of RAN formulations were determined using a Quant-it RiboGreen RNA assay kit (Thermo Fisher Scientific), and the cells were treated dose-dependently with each formulation at 10x desired dosage (20 μL into 180 μL of cells) and PBS as a control. After 24 h, supernatant was collected and a LumiKine Xpress 2.0 kit (InvivoGen) was used to measure secreted IFN-β per the manufacturer’s instructions. A nonlinear regression curve was fit to the data points using GraphPad Prism10.2.3 software.

#### 2.2.9 Cellular Viability

A CellTiter-Glo 3D cell viability luminescence-based assay (Promega) was utilized to report dose-dependent cytotoxicity of the system. Briefly, A549-Dual cells were seeded in 96-well clear TC-treated plates at a density of 5000 cells per well and allowed to adhere. Cells were then treated with nanoparticles at 10x desired dosage (20 μL into 180 μL of cells) and at 24 h were lysed with 30 μL of CellTiter-Glo reagent (Promega). After 20 min, 100 μL of supernatant from each well was then transferred to 96-well white-walled, opaque plates and immediately read for luminescence using a Synergy HI plate reader (Bio-Tek). Luminescent readouts were normalized to PBS control wells and reported as viability percentage. A nonlinear regression curve was fit to the data points, and IC_50_ values were obtained using GraphPad Prism10.2.3 software.

#### 2.2.10 Serum Stability

A549-Dual reporter cells were seeded at 5000 cells per well in 96-well, clear-bottom TC-treated plates and allowed to adhere overnight. Nanoparticles were formulated, and RNA concentrations were quantified as previously described. Formulations were then incubated with 50% FBS (Gibco) or PBS under gentle agitation at 37 °C for 4 h. Following incubation, the cells were treated at 10× desired dosage (20 μL into 180 μL of cells) with RAN formulations. After 24 h, interferon expression was measured on a Synergy HI plate reader (Bio-Tek) using a QUANTI-Luc luciferase-based detection assay (InvivoGen). Luminescent readouts for treated groups were normalized to PBS control wells and reported as relative luminescence (RLUs). A nonlinear regression curve was fit to the data points using GraphPad Prism10.2.3 software.

#### 2.2.11 Ex Vivo Analysis of Pro-Inflammatory Gene Expression

qRT-PCR analysis was performed to examine gene expression of pro-inflammatory markers at the tumor site. MC38 tumors were inoculated subcutaneously into the right flank of female six-week-old C57Bl/6J mice (Jackson Laboratory) at 1 × 10^6^ cells per mouse. Once tumors reached a volume of 100 mm^3^, mice were administered one intratumoral injection of RANs at a dose of 10 μg per mouse. At 4 h, mice were sacrificed and tumors were harvested and stored at −80 °C in RNAlater (Thermo Fisher Scientific) until used. For qRT-PCR, an RNeasy Plus Mini Kit (Qiagen) was used per manufacturer’s instructions. Briefly, ∼30 mg of tumors were weighed and suspended in 600 μL RLT lysis buffer, homogenized for 5 min, and centrifuged to pellet cellular debris. 350 μL of cell lysate was collected, and a QiaCube Connect (Version 1.2.1, Qiagen) was used to extract RNA using the RNeasy Plus Mini Kit protocol. Complementary DNA (cDNA) was synthesized for each sample using a cDNA synthesis kit (iScript, Bio-Rad), and a Taqman kit (Thermo Fisher Scientific) was used for the qRT-PCR reactions. Taqman probes (Thermo Fisher Scientific) for mouse Hmbs (Mm01143545_m1), mouse Cxcl9 (Mm00434946_m1), mouse Cxcl10 (Mm00445235_m1), and mouse Ifnb1 (Mm00439552_s1) genes were used. Fold-change was calculated using the ΔΔ*C*_t_ method.

#### 2.2.12 Tumor Site Cellular Uptake

MC38 tumors were inoculated subcutaneously on the right flank of female six-week-old C57BL/6 mice (Jackson Laboratory) as previously described. Following euthanasia, tumors were excised and dissociated using an Octomacs dissociator (Miltenyi Biotec). To break down tumors, they were placed in a tumor dissociation solution (Miltenyi Biotech) for 45 min at 37 °C shaking at 100 rpm and then dissociated again on an Octomacs dissociator. To obtain a single-cell suspension, tumors were mashed through a 70 μm strainer and then centrifuged at 380*g* for 5 min. After discarding the supernatant, cells were resuspended in 3 mL of ACK lysis buffer (KD Medical) for 5 min at room temperature. FC block was added for 15 min at 4 °C with surface stain added immediately after for 30 min at 4 °C. Cells were rinsed and centrifuged at 380*g* for 5 min, then fixed in 2% paraformaldehyde for 10 min at room temperature. Cells were resuspended in flow buffer (2% FBS in PBS + 0.05% sodium azide) and centrifuged at 650*g* for 5 min. Samples were run on an Aurora (Cytek) spectral flow cytometer and analyzed in Flowjo (BD Bioscience). Gating schematics are included in Figure S11.

#### 2.2.13 Antibodies

CD4 (RM4-5, BV605, Biolegend), CD44 (IM7, PerCP, Biolegend), CD366/Tim3 (RMT3-23, PE-Dazzle594, Biolegend), CD223/LAG3 (C9B7W, BV785, Biolegend), CD279/PD1 (29F.1A12, BV510, Biolegend), CD8α (53-6.7, AF488, Biolegend), CD69 (H1.2F3, PE-Cy7, Biolegend), CD62L (MEL-14, BV711, Biolegend) *K*_i_-67 (SolA15, AF532, ebioscience), Granzyme B (NGZB, PE-Cy5.5, ebioscience), KLRG1 (2F1, sb645, ebioscience), TCR-β (S33-966, ef450, ebioscience), and FoxP3 (FJK-16S, PE, ebioscience). TCRb­(H57-597, eFluor 450, eBioscience), CD4 (RM4-5, SB780, eBioscience), CD8a (53-6.7, BV605, BioLegend), CD11b (M1/70, BV510, BioLegend), CD11c (N418, BV711, BioLegend), GR-1 (RB6-8C5, PE/Cy7, eBioscience), F4/80 (BM8, AF488, BioLegend), and CD19 (6D5, PE, BioLegend).

#### 2.2.14 Tumor Site Retention Study

MC38 tumors were inoculated subcutaneously on the right flank of female six-week-old C57BL/6 mice (Jackson Laboratory) as previously described. Once tumors reached a volume of 50–100 mm^3^, mice were treated with 100 μL of RANs loaded with a 1:1 mixture of SLR14 and a fluorescently labeled SLR14 analog (SLR14-AF647) (10 μg/mouse). Live animal IVIS imaging was used to measure fluorescence longitudinally at various time points (i.e., 0 min, 15 min, 4 h, 8 h, 24 h, 48 h). After 48 h, mice were humanely euthanized. Percent initial average radiant efficiency values as a function of time were plotted and fit to a one-phase decay model (GraphPad Prism) which computed half-life measurements based on a rate constant (*k*) for each mouse.

#### 2.2.15 Subcutaneous MC38 and B16.F10 Tumor Models and Therapy Studies

MC38 (1 × 10^6^) cells per mouse or B16.F10 (5 × 10^5^) cells were injected subcutaneously into the right flank of female six-week-old C57BL/6 mice (Jackson Laboratory). Once tumors reached a volume of 50–100 mm^3^, mice were treated intratumorally with the following groups: RAN_2 kDa_-SLR14 (RAN) (10 μg/mouse, 100 μL), RAN_2 kDa_-SLR14-OH (cRAN) (10 μg/mouse, 100 μL), or PBS (100 μL) every 3 days for a total of 3 treatments. To evaluate therapeutic efficacy, tumor volumes were measured using calipers every 2 days, and volumes were calculated using (*V*_tumor_ = *L* × *W* ^2^ × 0.5). To assess toxicity, mice were monitored for weight loss throughout the course of the study. Mice were euthanized once tumors reached a volume of >1500 mm^3^ or they exhibited weight loss of >15%.

#### 2.2.16 Cell Culture

A549-Dual human lung adenocarcinoma cells (InvivoGen) were cultured in Dulbecco’s modified MEM (DMEM, Gibco) supplemented with 10% heat-inactivated fetal bovine serum (HI-FBS, Gibco), 100 U/mL penicillin (Gibco), 100 μg/mL streptomycin (Gibco), 2 mM l-glutamine, 25 mM HEPES (Invitrogen), 4.5 g/L d-glucose, and 100 μg/mL Normocin (InvivoGen). 100 μg/mL zeocin (InvivoGen) and 10 μg/mL blasticidin (InvivoGen) were added every other passage to maintain gene selection. MDA-MB-231 human breast carcinoma cells engineered to express a Gal8-YFP fusion protein were cultured in DMEM (Gibco) supplemented with 10% HI-FBS (Gibco), 100 U/mL penicillin (Gibco), 100 μg/mL streptomycin (Gibco), and 1× GlutaMAX (Gibco). B16.F10 murine melanoma cells and MC38 murine colon adenocarcinoma cells were cultured in DMEM supplemented with 10% HI-FBS (Gibco), 100 U/mL penicillin (Gibco), 100 μg/mL streptomycin (Gibco), 2 mM l-glutamine, 25 mM HEPES (Invitrogen), 4.5 g/L d-glucose, and 1 mM sodium pyruvate (Gibco). All cells were grown in a humidified atmosphere at 37 °C and 5% CO_2_. Cells were passaged with 0.05% Trypsin (Gibco) once ∼70% confluency was reached.

#### 2.2.17 Animal Care and Experimentation

Female six-week-old C57BL/6 mice were purchased from Jackson Laboratory (Bar Harbor, ME). All mice were housed and treated in compliance with regulations set forth by the Vanderbilt University Institutional Animal Care and Use Committee (IACUC).

#### 2.2.18 Statistical Analysis

GraphPad Prism 10 was used to analyze all data, and data is reported as mean ± SD or SEM. A ROUT test was utilized to identify outliers. Comparisons between two groups was conducted using an unpaired *t* test. For multiple comparisons, an ordinary one-way ANOVA analysis was conducted. To identify tumor volume significance in therapy studies, a two-way ANOVA analysis was conducted. Kaplan–Meier survival curves were analyzed by using a log-rank (Mantel-Cox) test. *P* values are depicted as *P < 0.05, **P < 0.01, ***P < 0.001, ****P < 0.0001.

## 3 Results and Discussion

### 3.1 Optimization of Flash Nanoprecipitation Process for RAN Fabrication

Our primary design considerations in the fabrication of RANs for intratumoral administration were to employ a facile, high-throughput, and industrially scalable process capable of efficiently packaging 3pRNA into uniform and stable polymeric nanoparticles that protect RNA cargo and promote endosomal escape into the cytosol. We postulated that this could be achieved by an FNP-based process in which a CIJ is used to induce turbulent micromixing between an aqueous stream of 3pRNA and an organic stream comprising a binary mixture of a corona-forming PEG-*b*-DB diblock polymer and core-forming DB chains. Upon impingement, the water-insoluble and endosomolytic DB polymer precipitates and provides cationic domains that electrostatically interact with anionic 3pRNA, forming a core that is surfactant stabilized by the PEG-*b*-DB corona-forming polymer, which provides aqueous solubility and colloidal stability. Although binary and ternary mixtures of polymers have been explored for siRNA and mRNA delivery applications,^42–44^ to our knowledge, CIJ mixing has yet to be utilized for polymeric delivery of immunostimulatory nucleic acids, such as RIG-I agonists. As previously described, we used RAFT polymerization to synthesize the core-forming DB polymer with a degree of polymerization (DP) of 100 (∼15 kDa), a size that has previously been demonstrated to promote endosomal escape.^42^ We also synthesized three corona forming PEG-*b*-DB polymers with mPEG first blocks of 2, 5, and 10 kDa, holding the second block molecular weight constant at 6 kDa (DP = 35). The PEG corona provides colloidal stability while simultaneously shielding the cationic and membrane interactive domains of DB which can contribute to carrier-induced toxicity. Because longer PEG chains and/or more dense PEG coronas can also impede cellular uptake following intratumoral injection, we evaluated three PEG molecular weights to explore potential differences in nanoparticle properties and activity. The second block of the corona-forming diblock copolymers is chemically identical to the core-forming DB polymer to promote favorable interactions and ensure analogous pH-responsive behavior. However, we synthesized these copolymers at a lower second block molecular weight (∼6 kDa) as to ensure that the self-assembly of the corona-forming polymer occurs on a time scale rapid enough to stabilize the nanoparticle core. Because the hydrophobic DB domains of the corona-forming polymers are miscible with the RAN core, a smaller hydrophobic block promotes the formation of a dense PEG surface to colloidally stabilize the nanoparticle.^45,46^ Additionally, such short 6 kDa DB chains are poorly endosomolytic,^42^ and therefore the endosomal escape properties of core–shell particles assembled via FNP can be primarily attributed to the core-forming polymer. The properties of DB and PEG-*b*-DB polymers are summarized in in Table 1 and polymer ^1^H NMR analysis is included in Figure S1. While PEG was used for the corona of RANs due to its well-established use in nanomedicines and approved therapeutics, there is a potential that RANs could raise anti-PEG antibodies or that pre-existing anti-PEG antibodies would compromise RAN efficacy. The RAFT polymerization process is highly versatile and amenable to synthesis to bespoke corona-forming surfactants that could be readily integrated into the RAN assembly process as an alternative to PEG.

We have previously used an FNP method to assemble pH-responsive polymersomes using chemically similar diblock copolymers and adapted this general approach to fabricate 3pRNA loaded NPs (Figure 1).^39^ We solubilized PEG-DB corona-forming polymers (PEG2 kDa, PEG5 kDa, PEG10 kDa) and core-forming polymer (DB) at desired ratios to a total of 1 mg/mL polymer in acetonitrile, which served as the organic inlet stream for CIJ mixing. The aqueous inlet stream was comprised of SLR14, a potent RIG-I agonist, solubilized in ultrapure distilled DNase-/RNase-free H_2_O. To fabricate RANs via FNP, the streams were impinged at a 50:50 solvent:antisolvent ratio into the CIJ mixer and collected in an aqueous reservoir of ultrapure distilled H_2_O under rapid stirring. In an attempt to reduce polymer-associated toxicity^47^ and improve cargo and carrier stability, our initial screen sought to minimize the polymer to RNA inlet ratios required for maximal cargo encapsulation while maintaining desirable nanoparticle properties, such as size and uniformity. We fabricated RANs at three ratios corresponding to 4:1, 8:1, and 12:1 nitrogen to phosphate ratios (N:P) at a 1:1 ratio (mass) of corona-forming polymer to core-forming polymer. Dynamic light scattering (DLS) was used to measure particle size and polydispersity (PDI) and a Quant-it RiboGreen RNA assay was used to measure RNA loading efficiency (Figure 2A). While no significant deviations in nanoparticle size and sample polydispersity were evident between groups, we noticed significant improvement in loading efficiency at the higher N:P ratios tested (8:1 and 12:1) when compared to the lower N:P ratio (4:1). As a result, we chose to proceed with the 8:1 N:P ratio for the remainder of the studies, as this formulation allowed for the highest loading efficiency at the lowest polymer:RNA ratios. To further elucidate the effect of each polymer component on particle properties, we implemented organic inlet feeds comprised predominantly of core-forming polymer (3:1 core:corona) and predominantly of corona-forming polymer (1:3 core:corona) (Figure S2). It became evident that RANs fabricated at the 8:1 N:P ratio allowed for some of the highest encapsulation efficiencies with each core:corona ratio tested. While we noticed slight improvement in sample uniformity (lower PDI) for the 3:1 group at this N:P ratio, no significant improvement in encapsulation efficiency was evident, and RANs exhibited an ∼2-fold increase in size. Ultimately, this finding highlights the tunability of this approach to produce immunostimulatory nanoparticles with structural properties favorable for various delivery applications; however, we chose to proceed with the 1:1 core:corona inlet ratio at the 8:1 N:P ratio, as it offered superior loading efficiencies (>85%) and a particle size range (∼100 nm) commonly reported for both local and systemic delivery applications of RIG-I agonists.^30,34,48^

**Figure 2:**
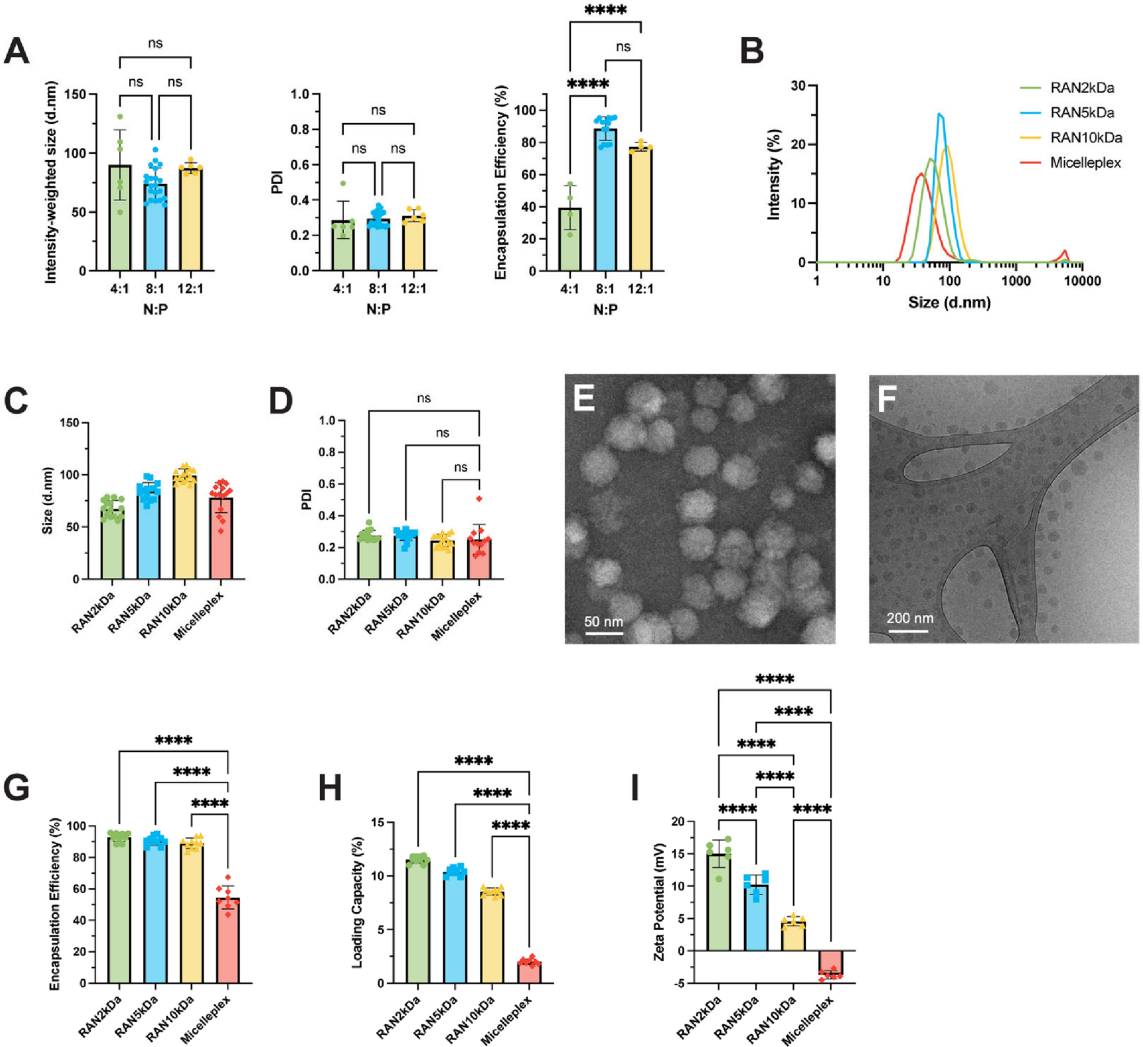
Parameter optimization and characterization of RAN properties. (A) Initial screen for RAN size, polydispersity index (PDI), and encapsulation efficiency (EE) as a function of nitrogen to phosphate (N:P) ratio in the inlet CIJ streams (*n* = 2–5 experimental replicates per group) *P* values determined by an ordinary one-way ANOVA test with Tukey’s test for multiple comparisons. (B) Dynamic light scattering (DLS) intensity-weighted size distributions, (C) diameter, and (D) PDI for each RAN formulation (*n* = 4–5 experimental replicates per group). *P* values determined by an ordinary one-way ANOVA test with Dunnett’s test for multiple comparisons. (E) Representative transmission electron microscopy (TEM) images (scale bar = 50 nm) and (F) cryogenic electron microscopy (cryoEM) images (scale bar = 200 nm) of RAN_2 kDa_ formulation. Assembly of RANs with FNP results in significantly higher (G) EEs and (H) loading capacities (LCs) than electrostatic complexation to form micelleplexes at the same N:P ratio of 8:1 (*n* = 4–5 experimental replicates per group). *P* values determined by an ordinary one-way ANOVA test with Dunnett’s test for multiple comparisons. (I) Zeta potential measurements (*n* = 2 experimental replicates per group). *P* values determined by an ordinary one-way ANOVA test with Tukey’s test for multiple comparisons. Replicates are experimental and technical, and data are shown as mean ± SD. **** signifies *P* < 0.0001.

We next sought to examine whether the self-assembly of RANs was dependent on the nucleic acid therapeutic or if this self-assembly was driven mainly by hydrophobic interactions during the flash nanoprecipitation process. To assess this, we fabricated empty RANs and used DLS to measure nanoparticle diameter and sample polydispersity. While minor differences in diameter (<20 nm) and PDI (<0.05) were observed between loaded and empty RANs, the empty nanoparticles were still able to self-assemble in the absence of a nucleic acid cargo (Figure S3). Note that to conserve valuable 3pRNA for these studies, we used an analogous control SLR hairpin that lacks the 3p group and instead bears a 5′-OH group (SLR14-OH). One of the major benefits of FNP is the scalability of the process, with a wide range of inlet polymer concentrations being reported in the literature for various applications.^39,49–52^ Although we implemented a base 1 mg/mL inlet polymer concentration for this study to conserve SLR14 cargo and minimize polymer:RNA ratios, we also fabricated empty RANs at increasing inlet polymer concentrations to examine the scalability of our core–shell nanoparticle platform in a cargo-agnostic manner. We observed a direct correlation between inlet polymer concentration and nanoparticle size (Figure S4A,B). We also noticed a slight improvement in sample uniformity at some of the higher inlet polymer concentrations (5 and 10 mg/mL) (Figure S4C). Together, these findings suggest that FNP facilitates the formation of tunable nanocarriers in a cargo-agnostic manner, warranting further exploration of their potential for loading other immunotherapeutics including proteins, mRNAs, and siRNAs.

Using the established inlet conditions from the aforementioned study, we next investigated the effect of PEG length of the corona-forming diblock copolymer on nanoparticle physical properties. RANs were fabricated and screened for properties including size, polydispersity, surface charge, and RNA encapsulation efficiency. As a control, we also used our previously described formulation based on micelleplexes, formed by electrostatic loading of 3pRNA (here, SLR14) into PEG_10 kDa_-*bl*-DB_25 kDa_ diblock polymers at acidic pH followed by neutralization.^35^ To allow for comparison with FNP formulation, we used the same 8:1 N:P ratio for micelleplex assembly. In evaluating particle size and uniformity, intensity-weighted DLS indicated unimodal size distributions for RAN_2 kDa_, RAN_5 kDa_, and RAN_10 kDa_ nanoparticles (Figure 2B), and an increase in the intensity-weighted size was observed with the larger PEG block in the corona-forming polymer, while insignificant changes in polydispersity were observed (Figure 2C,D). Additionally, all RANs were found to be colloidally stable in sterile PBS at 4 °C storage conditions for at least 3 weeks, ideal for large-scale batch processes common in pharmaceutical manufacturing (Figure S5). Transmission electron microscopy (TEM) (Figures 2E and S6A,B) and cryogenic electron microscopy (cryoEM) imaging (Figures 2F and S6C,D) of RANs revealed spherical particles with sizes comparable to that measured by DLS and a solid core consistent with nanoprecipitation of DB and SLR14. Importantly, we found that the FNP process allowed for near quantitative loading of SLR cargo, with ∼90% encapsulation efficiency and ∼10% loading capacity achieved; by contrast, our previous micelleplex approach was associated with ∼50% SLR encapsulation efficiency and ∼2.5% loading capacity at the same polymer:RNA ratio (Figure 2G,H). In our previous work, we demonstrated that an N:P ratio of ∼30 was required to load a 22-mer dsRNA molecule into PEG_10 kDa_-*bl*-DB_25 kDa_ micelleplexes with >80% efficiency.^35^ Collectively, these results highlight the effectiveness of FNP in encapsulating greater amounts of SLR with lower required polymer inputs, thereby minimizing cargo loss during the formulation process and reducing the risk of polymer-associated toxicities. All RANs assembled by FNP had a positive zeta potential that decreased with increasing PEG molecular weight, indicating that longer PEG chains were more effective in shielding the cationic DMAEMA groups in the core (Figure 2I). Interestingly, micelleplexes possessed a slightly negative surface charge, most likely indicative of loose and inefficient RNA complexation and surface exposure of phosphate groups when loaded at this lower N:P ratio. This finding indicates that the use of the FNP process to form core–shell particles from a binary polymer blend is more efficient at RNA loading than electrostatic-driven assembly of micelleplexes, resulting in a ∼2-fold reduction in the total amount of polymer used and a ∼1.6-fold reduction in the cationic DB component, which contributes to the cytotoxicity of these and other polycationic drug carrier systems. Therefore, with just a single induction of turbulent mixing, we find that FNP can produce uniform, nucleic acid-loaded polymeric nanoparticles on the time scale of milliseconds, a method that also offers tunability and scalability amenable to a large-scale pharmaceutical manufacturing process. Additionally, this process has the potential to reduce processing times and variability commonly faced with other methods for loading nucleic acid cargos within nanoparticles, such as manual mixing, emulsion, or pH-driven self-assembly mechanisms.

### 3.2 RANs Induce Potent Endosomal Disruption and Immunostimulatory Activity In Vitro

Critical to the effectiveness of this drug delivery platform is sufficient endosome-destabilizing activity to release the cargo into the cytosol. We first assessed this in vitro by conducting a galectin 8 (Gal 8) recruitment reporter assay to evaluate particle-induced endosome disruption.^53^ MDA-MB-231 human breast adenocarcinoma cells engineered to stably express a Gal8-yellow fluorescent protein (Gal8-YFP) fusion protein were treated with RANs and incubated overnight. Upon endosomal disruption, Gal8-YFP proteins dispersed throughout the cytosol will bind to exposed glycans within the ruptured endosome in the form of distinct fluorescent puncta (Figure 3A). The cells are imaged with fluorescent microscopy, and an image processing algorithm is used to calculate the number of fluorescent puncta per cell, which has been shown to correlate with the degree of endosomal disruption.^53,54^ RANs were loaded with an inactive SLR14-OH analog to ensure that any signal was a result of endosomal disruption and not an indirect effect of RIG-I activation. After treatment and incubation for 24 h, it was determined that all RAN formulations induced Gal8-YFP puncta formation in a dose-dependent manner, with EC50 values in the 1–4 μM range (based on polymer dose) (Figure 3B,C). RAN_2 kDa_ and RAN_5 kDa_ nanoparticles were more active compared to RAN_10 kDa_ nanoparticles (Figure 3C), as indicated by a ∼4× lower EC50, most likely due to increased PEG shielding and decreased zeta potential that reduced intracellular uptake and/or interactions with the endosomal membrane.

**Figure 3:**
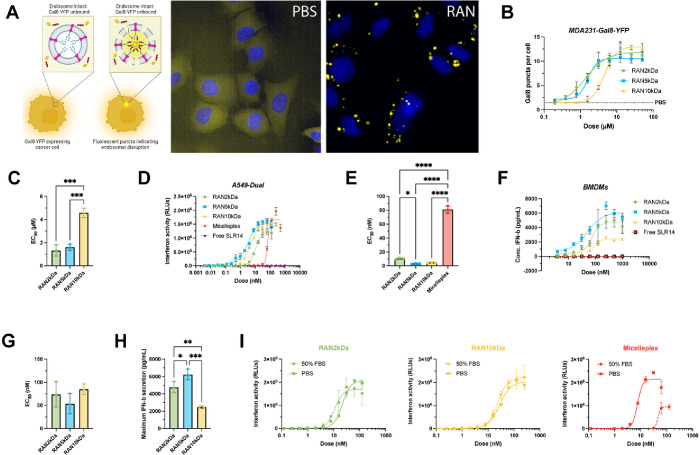
RANs are endosomolytic, immunostimulatory in vitro, and stable in serum. (A) Experimental schematic of Gal8-YFP assay used to measure endosomolytic activity of RANs and representative microscopy images of MDA-MB-231 Gal8-YFP cells treated with a RAN formulation and a PBS control. (B) Dose response curves of MDA-MB-231 Gal8-YFP cells treated with indicated RAN formulations (*n* = 3 biological replicates per group). All RANs promoted endosomolytic activity in a dose-dependent manner as indicated by (C) calculated EC_50_ values of each RAN formulation. *P* values determined by an ordinary one-way ANOVA test with Tukey’s test for multiple comparisons. (D) Dose response curves for relative IFN-I production by A549-Dual reporter cells treated with RAN formulations, micelleplex, and free SLR14 controls (*n* = 3 biological replicates per group). (E) Calculated EC_50_ values for relative IFN-I production. *P* values determined by an ordinary one-way ANOVA test with Dunnett’s test for multiple comparisons. (F) Dose response curves for IFN-β secretion by isolated bone marrow-derived macrophages (BMDMs) treated with RAN formulations (*n* = 2 biological replicates per group). (G) Calculated EC_50_ values and (H) maximum secreted IFN-β levels. *P* values determined by an ordinary one-way ANOVA test with Tukey’s test for multiple comparisons. (I) Serum stability analysis of RAN_2 kDa_ (green), RAN_10 kDa_ (yellow), and micelleplex (red) immunostimulatory activity in A549-Dual IFN-I reporter cells after 24 h incubation in either 50% FBS or PBS at 37 °C. Replicates are biological, and data are shown as mean ± SD * signifies *P* < 0.05, ** signifies *P* < 0.01, *** signifies *P* < 0.001, **** signifies *P* < 0.0001.

To assess the ability of RANs to induce potent RIG-I activation in vitro, we next examined immunostimulatory activity in A549-Dual (human lung carcinoma) cells engineered with an interferon regulatory factor (IRF)-inducible luciferase reporter which allows for a relative quantification of type-I interferon (IFN-I) secretion when compared to untreated cells. We found that all three RAN formulations enhanced IFN-I pathway activation with similar potency in a relevant cancer cell line (Figure 3D) and that all RAN formulations stimulated RIG-I activation at significantly lower doses than the micelleplex formulation, as reflected in the corresponding EC_50_ values (Figure 3E); as expected, free 3pRNA did not exhibit discernible activity. Additionally, we found no significant differences between toxicity profiles in A549-Dual cells treated with each RAN formulation (Figure S7). Notably, cell viability is high within the dose range near the EC_50_ of each carrier, indicative of immunostimulatory activity with minimal toxicity. As a benchmark, we also compared the activity of RANs to an analogous RAN formulation loaded with polyIC, an established RIG-I agonist that has advanced to clinical trials for intratumoral immunotherapy.^55,56^ Although both formulations displayed similar encapsulation efficiencies (∼90%), RANs loaded with SLR14 induced an interferon-dependent immune response in A549-Dual reporter cells at significantly lower doses than RANs loaded with polyIC, as reflected in the corresponding EC_50_ values (Figure S8A–C). We further screened RANs in these cells by implementing control groups of empty RANs and RANs loaded with an inactive hydroxylated SLR14 cargo lacking the 3p- group (SLR-OH) to ensure that interferon activity in these cells was a direct result of SLR14 activating RIG-I. As expected, we observed negligible activity for each of these control groups, confirming dependence on RIG-I activation (Figure S9). To assess RAN activity in primary myeloid cells relevant to the tumor microenvironment, murine bone marrow-derived macrophages (BMDMs) were harvested, plated, and treated with each RAN formulation, and IFN-β secretion levels were measured via ELISAs. All RAN formulations were found to increase IFN-β production with similar EC_50_ values (Figure 3F,G), though RAN_2 kDa_ and RAN_5 kDa_ elicited significantly higher maximum levels of secreted IFN-β relative to RAN_10 kDa_ (Figure 3H). We postulate that this is consistent with the Gal8 reporter assay in which RAN_2 kDa_ and RAN_5 kDa_ nanoparticles were more potently endosomolytic than RAN_10 kDa_ nanoparticles at equivalent dosages, reinforcing the importance of endosomal escape promoting cytosolic delivery and mitigating 3pRNA degradation within the harsh acidic environment of the late endosome and early lysosome.^57,58^

An optimally designed nanoparticle should shield the cargo from nucleases within serum or other biological fluids, preserving 3pRNA structure for oriented RIG-I receptor binding and maintenance of immunostimulatory activity. This is particularly important for 3pRNA since backbone modifications used to stabilize siRNA therapeutics, such as 2′-F or phosphorothioate modifications,^59^ have not been widely explored for stabilization of 3pRNA. Furthermore, these backbone modifications are likely to require substantial optimization due to complex molecular interactions with RIG-I. To assess the stability of RANs in serum, we incubated samples in either 50% FBS or PBS for 24 h and then immediately evaluated their activity in A549-Dual reporter cells. We observed no significant losses in activity for any RAN formulation after serum incubation, indicating that polymer carriers were able to effectively protect the RNA cargo from nucleases and other proteins they might interact with in the serum. Interestingly, micelleplexes were found to lose their immunostimulatory effects when incubated with 50% FBS for the same amount of time (Figure 3I), consistent with our previous findings.^35,60^ Collectively, these findings indicate that assembly of RANs via FNP is highly effective at loading 3pRNA into the particle core and protecting it from degradation, resulting in increased immunostimulatory activity and improved serum stability relative to the micelleplex formulation.

### 3.3 Intratumoral Administration of RANs Stimulates a Localized Innate Immune Response

We next sought to evaluate the immunostimulatory activity of RANs when administered via an intratumoral route. For these studies, we used a subcutaneous MC38 murine colon adenocarcinoma model commonly employed in preclinical immunotherapy development,^61,62^ as this model has previously been demonstrated to respond to local administration of RIG-I agonists and other intralesional therapies (e.g., oncolytic viruses).^63–67^ Mice with subcutaneous MC38 tumors (∼100 mm^3^) were administered RANs intratumorally at doses corresponding to 10 μg/mouse SLR14, tumors were isolated 4 h following treatment, and the expression of pro-inflammatory markers characteristic of RIG-I activation was analyzed via qRT-PCR. While PEG molecular weight had relatively modest impact on RAN properties and activity in vitro, we nonetheless compared the activity of RANs assembled using 2 and 10 kDa PEG coronas. It was found that intratumoral treatment with both RAN formulations significantly upregulated expression of pro-inflammatory markers—*Cxcl9*, *Cxcl10*, and *Ifnb1*—to a comparable extent compared to PBS controls (Figure 4A), consistent with immunostimulation via RIG-I signaling.

**Figure 4:**
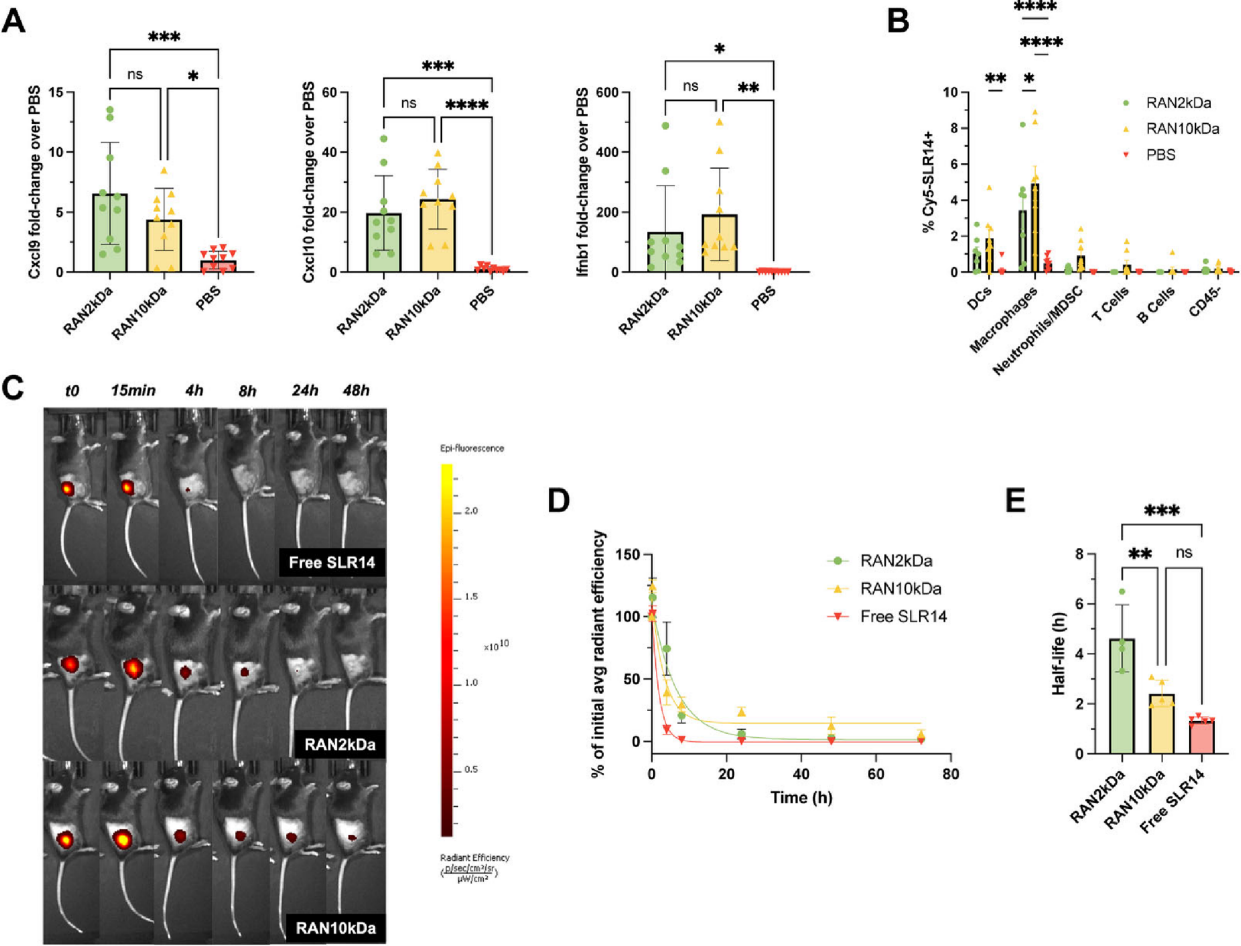
RAN formulations are endocytosed by tumor-associated myeloid cells and stimulate innate immunity following intratumoral administration. (A) RAN formulations upregulate RIG-I-driven proinflammatory markers compared to PBS vehicle controls in MC38 tumors 4 h after one intratumoral treatment (*n* = 10 mice per group). *P* values determined by an ordinary one-way ANOVA test with Dunnett’s test for multiple comparisons. (B) Cellular uptake of Cy5-SLR14-OH cargo by indicated cell populations as determined by flow cytometry 4 h after intratumoral administration of RANs in MC38 tumor model (*n* = 7–9 mice per group). (C, D) Intratumorally administered RANs increase the retention time of SLR14 within the tumor vasculature compared to free drug. (E) RANs increase the half-life of SLR14 compared to free drug (*n* = 4–5 mice per group). *P* values determined by a two-way ANOVA test with Tukey’s test for multiple comparisons. Replicates are biological, and data are shown as mean ± SD * signifies *P* < 0.05, ** signifies *P* < 0.01, *** signifies *P* < 0.001, **** signifies *P* < 0.0001.

To better understand the primary cellular contributors to this response, we evaluated the cellular uptake profile of RANs in MC38 tumors 4 h after intratumoral administration. Mice were sacrificed, tumors were harvested and dissociated, and flow cytometry was performed to determine the frequency of major cell populations positive for a fluorescently labeled SLR analog (Cy5-labeled SLR-OH) that was loaded into RANs via FNP using the same conditions. SLR14 was found to be mainly taken up by CD11b^+^F4/80^+^ macrophages and CD11c^+^ dendritic cells, albeit in only <5–10% of these cell types (Figure 4B). Interestingly, RAN_10 kDa_ nanoparticles exhibited slightly higher uptake across all cell types. We hypothesized that this could be a synergistic effect of the slightly cationic surface charge enhancing electrostatic interactions with cellular membranes, while the more heavily PEGylated surface may reduce rapid tumor clearance and/or improve perfusion throughout the tumor. To evaluate this, mice with subcutaneous MC38 tumors were intratumorally administered RANs loaded with a fluorescently labeled SLR14 analog, and IVIS imaging was used to monitor cargo retention over time (Figure 4C). As expected, RANs prolonged the retention of SLR14 within the tumor compared to free drug. Interestingly, while RAN_10 kDa_ was initially more rapidly cleared, residual signal was observed at later time points when compared to RAN_2 kDa_ (Figure 4D). We believe that this is a combined result of the more heavily PEGylated corona and larger size of RAN_10 kDa_ causing the formulation to be retained in the tumor microenvironment for longer. The biological significance of this small amount of persistent SLR14 is likely minimal and may also reflect inactive cargo as SLR may be partially or completely degraded by tumor-associated proteases, diminishing its immunostimulatory activity. Based on these findings, we chose to proceed with our RAN_2 kDa_ formulation for further in vivo analysis, as it displayed a significantly longer half-life than both RAN_10 kDa_ and free SLR14 (Figure 4E).

### 3.4 Intratumoral Administration of RANs Enhances Antitumor Efficacy in Murine Colon Cancer and Melanoma Models

Finally, we assessed the ability of RANs to mitigate disease progression in the same subcutaneous MC38 disease model. Based on their similar physicochemical properties, retention at the tumor site, and in vivo activity, we selected RAN_2 kDa_ for these studies. Once tumors reached a volume of 100 mm^3^, mice were given three intratumoral injections (10 μg/mouse), 3 days apart, and tumor growth and survival were monitored based on a humane end point of a tumor volume of >1500 mm^3^ (Figure 5A). Previously, we have shown therapeutic efficacy in subcutaneous MC38 models after intratumoral treatment with SLR14 at a dosage of 25 μg/mouse using jetPEI as a delivery vector.^17^ In this work, we wished to see if our RAN platform could allow for us to drop the SLR14 dose required to see therapeutic efficacy. At a dose of 10 μg/mouse, we found that RANs significantly reduced tumor burden and prolonged survival in mice compared to PBS controls as well as RANs formulated with an inactive SLR14 cargo lacking the 3p group (control RAN, cRAN), confirming a dependence on RIG-I activation for antitumor efficacy (Figure 5B–D). Additionally, no significant weight loss was observed post-treatment, indicating minimal, if any, treatment-related systemic toxicity (Figure 5E). We also examined the efficacy of RANs in a B16.F10 murine melanoma model, which is widely considered to be poorly immunogenic (i.e., “cold”) and resistant to immune checkpoint inhibitors.^68^ Mice with B16.F10 melanoma tumors on the right flank were treated with three intratumoral injections, 3 days apart (Figure 5F). Again, RANs significantly slowed tumor growth rate and prolonged survival compared to PBS controls (Figure 5G,H); additionally, no notable weight loss indicative of systemic toxicity was observed in mice upon RAN treatment (Figure S10A). While additional investigation is necessary to understand the mechanisms underlying efficacy, these studies position RANs as a promising technology for intratumoral immunotherapy.

**Figure 5:**
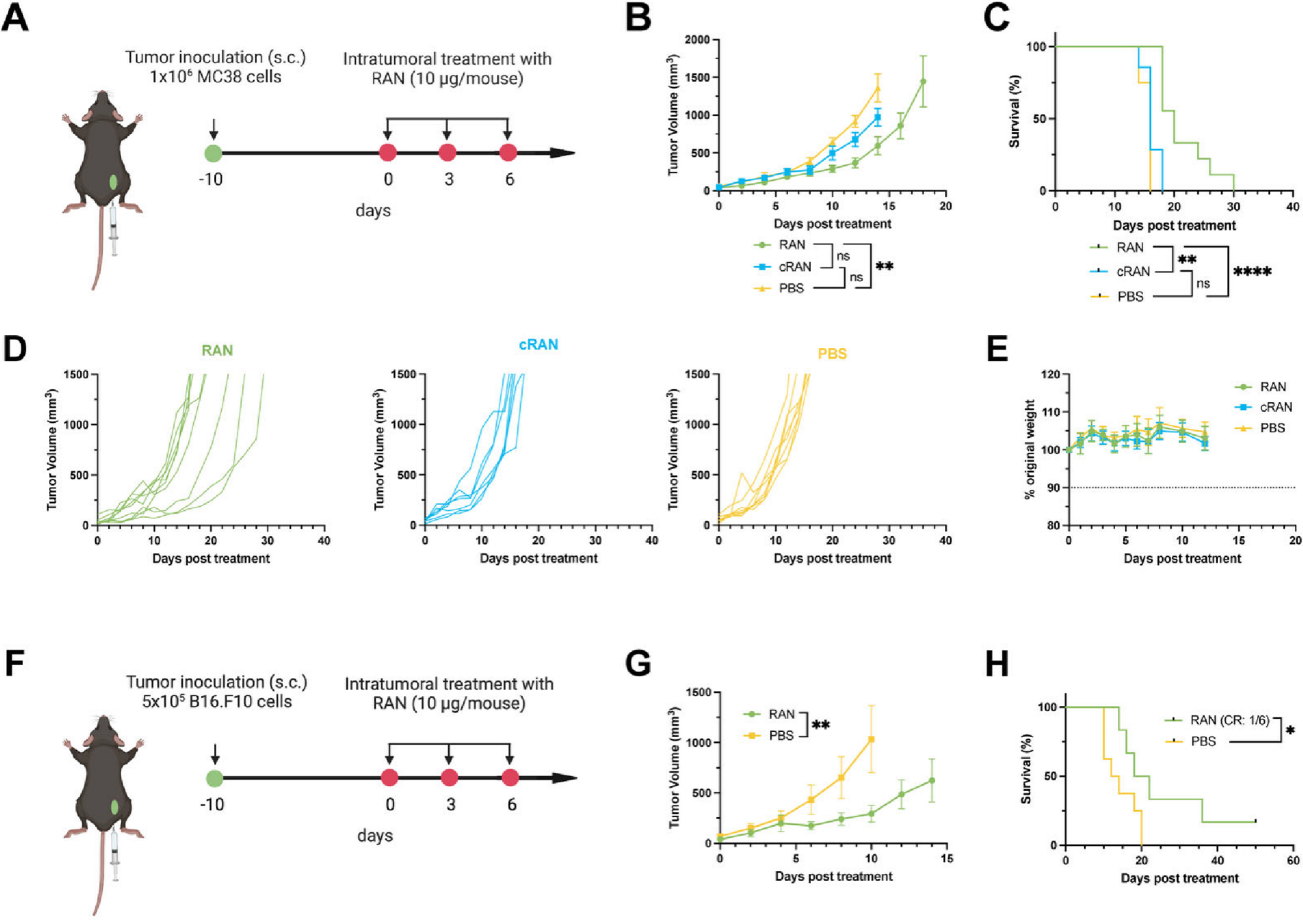
RANs mitigate disease progression and prolong survival in murine cancer models. (A) Experimental timeline and treatment schedule for intratumoral administration of mice with subcutaneous MC38 tumors. (B) Average tumor growth curves and (C) Kaplan–Meier survival plots for mice with MC38 tumors treated with indicated formulations. *P* values for tumor growth curves determined by a two-way ANOVA test with Tukey’s test for multiple comparisons on Day 14 shown. Survival curve comparisons were made using a Log-rank (Mantel-Cox) test. (D) Spider plots of individual tumor growth curves. (E) No notable weight loss was observed over the course of treatment (*n* = 7–9 mice per group). (F) Experimental timeline and treatment schedule for intratumoral administration of mice with subcutaneous B16.F10 tumors. (G) Average tumor growth curves and (H) Kaplan–Meier survival plots for mice with B16.F10 tumors treated with indicated formulations; CR = complete responder (*n* = 6–8 mice per group). *P* values determined by a two-way ANOVA test with Tukey’s test for multiple comparisons on Day 10 shown. Survival curve comparisons were made using a Log-rank (Mantel-Cox) test. Replicates are biological, and data are shown as mean ± SEM * signifies *P* < 0.05, ** signifies *P* < 0.01, **** signifies *P* < 0.0001.

## 4 Conclusions

Retinoic acid-inducible gene I (RIG-I) agonists are an exciting class of therapeutics with immense potential for stimulating antitumor innate immunity. However, their clinical translation has been limited in part due to their poor druglike properties that hinder cellular uptake and cytosolic bioavailability. To address this, we have developed RIG-I-activating nanoparticles (RANs)—a polymeric nanoparticle-based delivery platform for cytosolic delivery of triphosphorylated RNA (3pRNA) cargos. RAN assembly was achieved via a flash nanoprecipitation (FNP) process that enabled rapid and turbulent micromixing between an organic stream, which contained a blend of surface- and core-forming endosomolytic polymers, and an aqueous stream, which contained the 3pRNA therapeutic. By evaluating the effect of inlet stream ratios and/or PEG corona molecular weight, we generated a series of RANs that were <100 nm in diameter, encapsulated 3pRNA at >85% efficiency, promoted endosomal escape, exhibited high stability in serum, and potently stimulated innate immunity. Furthermore, we demonstrated that this formulation process enables improved RNA loading and increased immunostimulatory activity relative to a previously described micelleplex assembly method. Based on these favorable properties, we tested the efficacy of intratumorally administered RANs in MC38 colon cancer and B16.F10 melanoma models, finding that RANs were able to significantly mitigate tumor growth in a RIG-I-dependent manner. This positions RANs as a promising technology for intralesional therapy and highlights the use of FNP as a versatile, tunable, and scalable method for fabrication of RNA-loaded polymeric nanoparticles with potential utility for improving delivery of other classes of nucleic acid therapies, including siRNA, mRNA, and DNA.

## Supplementary Material



## Notes

Corona-forming and core-forming polymer structures; ^1^H NMR characterization of corona-forming polymer; ^1^H NMR characterization of core-forming polymer; core-forming:corona-forming polymer inlet ratio optimization; physical characterization of empty RANs; inlet polymer concentration scale-up analysis; long-term colloidal stability analysis; additional transmission electron microscopy (TEM) and cryogenic electron microscopy (cryoEM) images of RANs; in vitro toxicity in A549-Dual cells; in vitro activity in A549-Dual cells of RANs loaded with polyIC; in vitro activity in A549-Duals of empty RANs and control RANs; weight loss curves and spider plots for B16.F10 melanoma therapy study; and flow cytometry gating schematics for uptake study in MC38 tumor microenvironment (PDF)
