# Supplementary material for: Fabrication of RIG-I-Activating Nanoparticles for Intratumoral Immunotherapy via Flash Nanoprecipitation
Source: Mol Pharm. 2025 Jul 1;22(8):4597–611. doi: 10.1021/acs.molpharmaceut.5c00125 (PMC12338309; doi:10.1021/acs.molpharmaceut.5c00125)
Supplement: Supplementary file 1 [file mp5c00125_si_001.pdf]

Electronic Supplementary Information

Fabrication of RIG-I-Activating Nanoparticles for Intratumoral Immunotherapy via Flash Nanoprecipitation

Payton T. Stone[1], Alexander J. Kwiatkowski[1,2], Eric W. Roth[3], Olga Fedorova[4,5], Anna M. Pyle[4,5,6], John T. Wilson[1,2,7-11*]

[1]Department of Chemical and Biomolecular Engineering, Vanderbilt University, Nashville, TN 37235, United States.
[2]Department of Biomedical Engineering, Vanderbilt University, Nashville, TN 37235, United States.
[3]NU*ANCE* BioCryo, Northwestern University, Evanston, IL 60208, United States.
[4]Department of Molecular, Cellular, and Developmental Biology, Yale University, New Haven, CT 06520, United States.
[5]Howard Hughes Medical Institute, Chevy Chase, Maryland 20815, United States.
[6]Department of Chemistry, Yale University, New Haven, Connecticut 06520, United States.
[7]Vanderbilt Center for Immunobiology, Vanderbilt University Medical Center, Nashville, TN 37232, United States.
[8]Vanderbilt Institute for Infection, Immunology, and Inflammation, Vanderbilt University Medical Center, Nashville, TN 37232, United States.
[9]Vanderbilt Institute of Chemical Biology, Vanderbilt University, Nashville, TN 37232, United States.
[10]Vanderbilt Institute of Nanoscale Science and Engineering, Vanderbilt University, Nashville, TN 37232, United States.
[11]Vanderbilt-Ingram Cancer Center, Vanderbilt University Medical Center, Nashville, TN 37232, United States.

*Corresponding Author: john.t.wilson@vanderbilt.edu

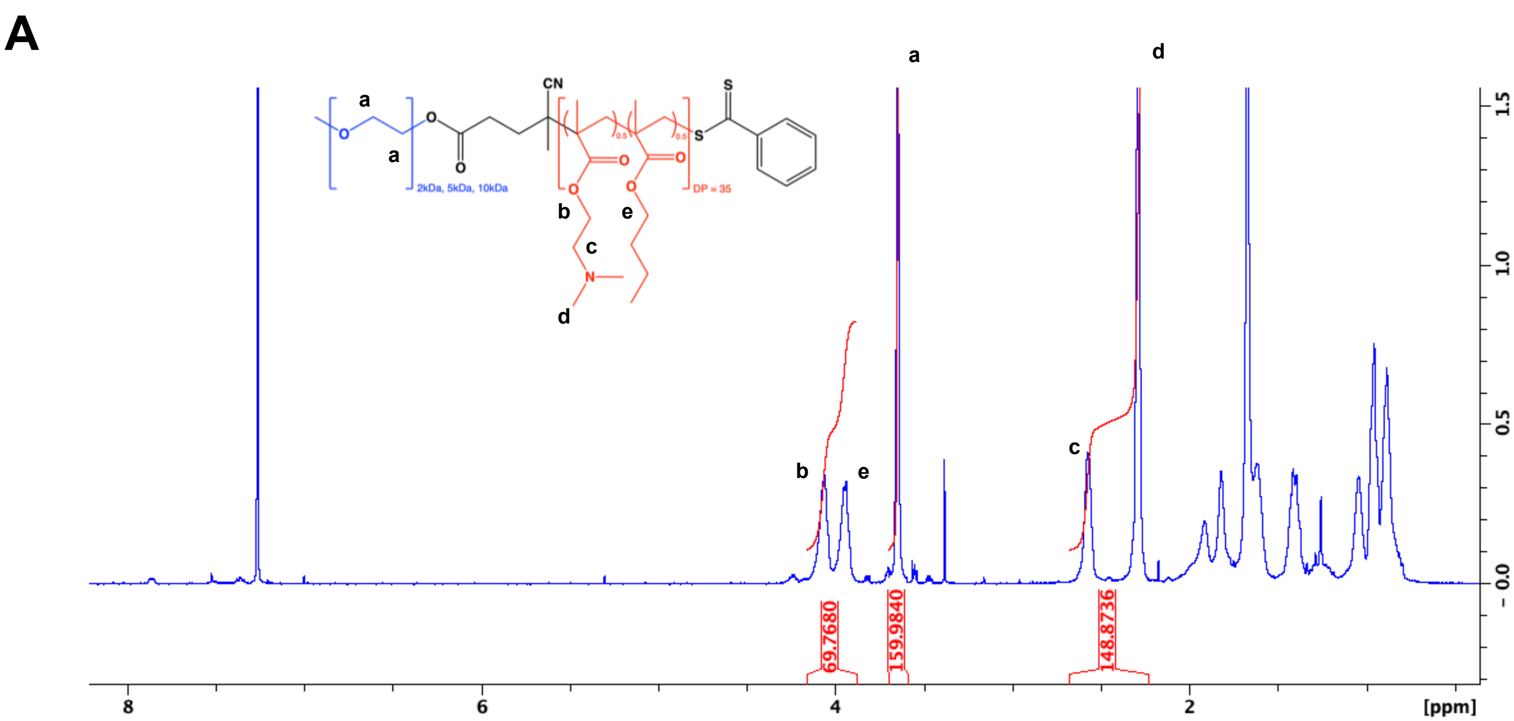

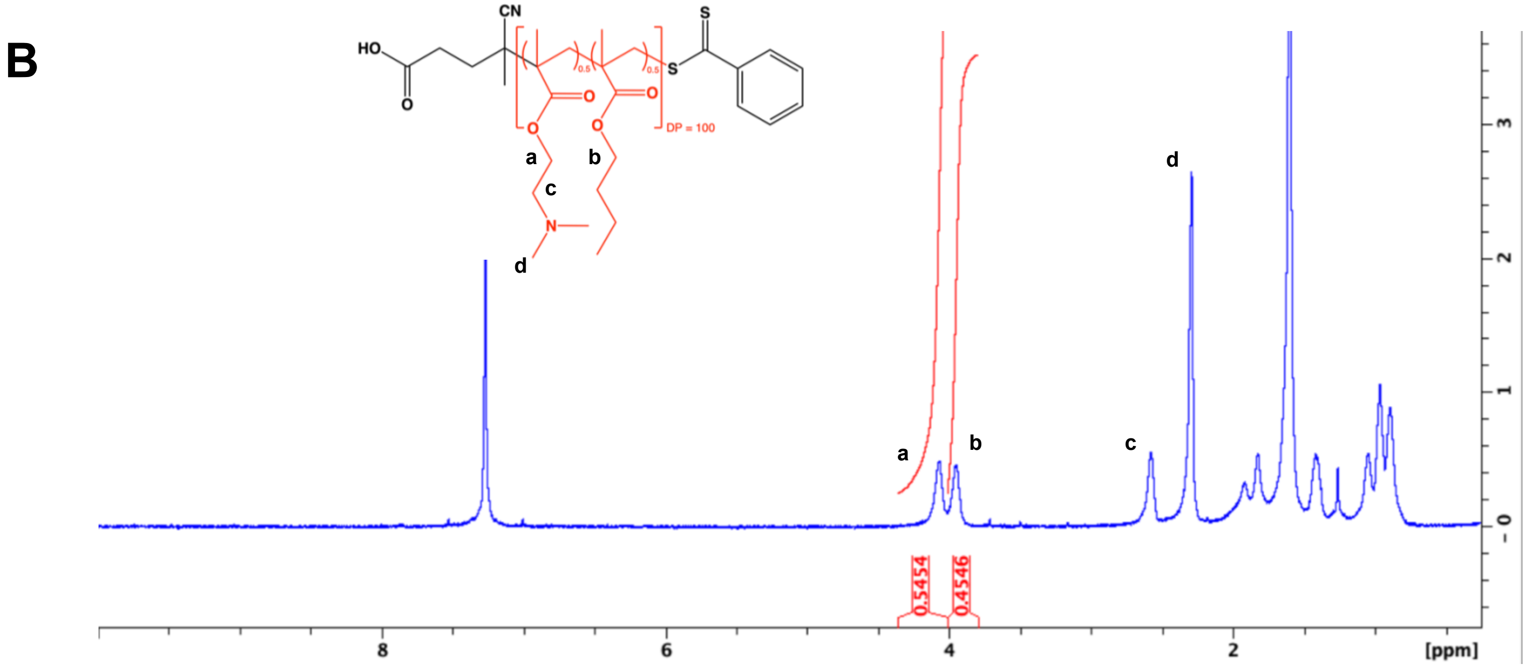

Figure S1. Polymer library characterization. (**A**) Representative ^{1}H-NMR analysis ($CDCl_3$) of PEG-*b*-[DMAEMA-co-BMA] corona-forming diblock copolymer, and (**B**) representative ^{1}H-NMR analysis ($CDCl_3$) of (DB) core-forming polymer. ^{1}H-NMR analysis conducted with Bruker TopSpin 3.6.3. Polymer structures created with ChemDraw 20.1.0.112.

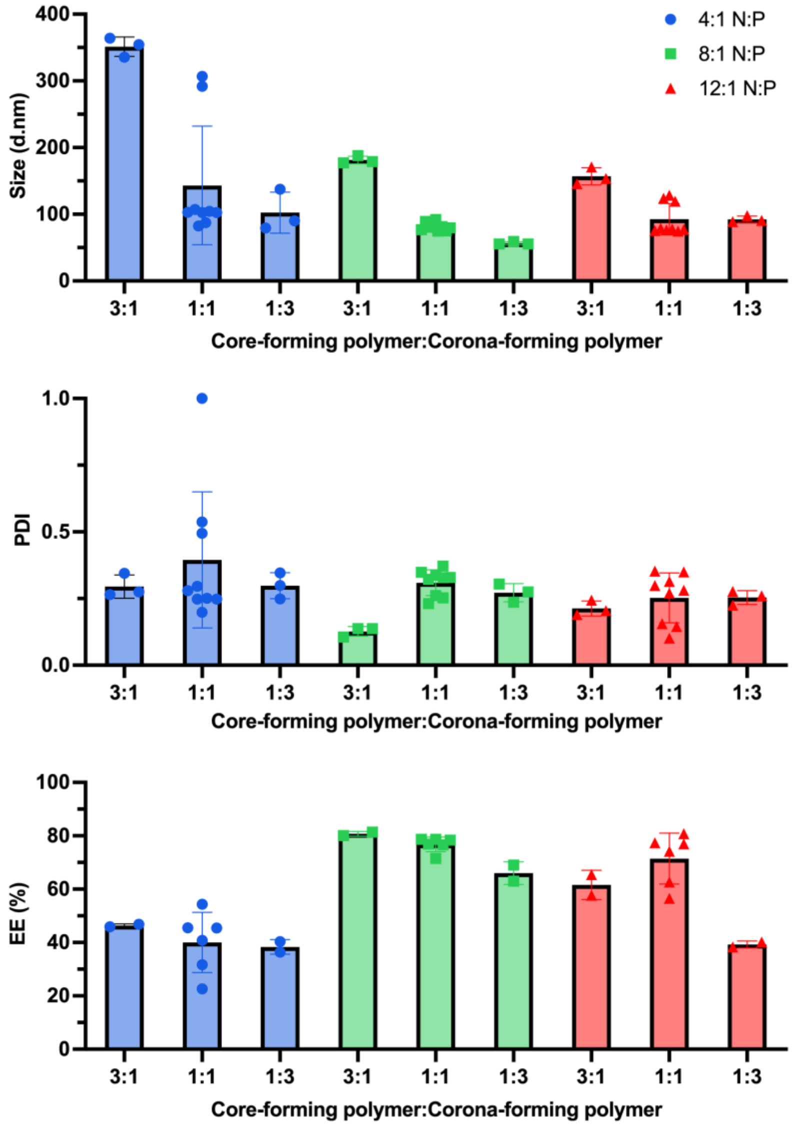

Figure S2. Core-forming to corona-forming polymer inlet ratio sweep. Size, polydispersity, and encapsulation efficiency measurements for RANs fabricated at core-forming:corona:forming polymer mass ratios (1:3, 1:1, and 3:1) and N:P ratios (4:1, 8:1, and 12:1). Replicates are experimental, and data are shown as mean ± SD.

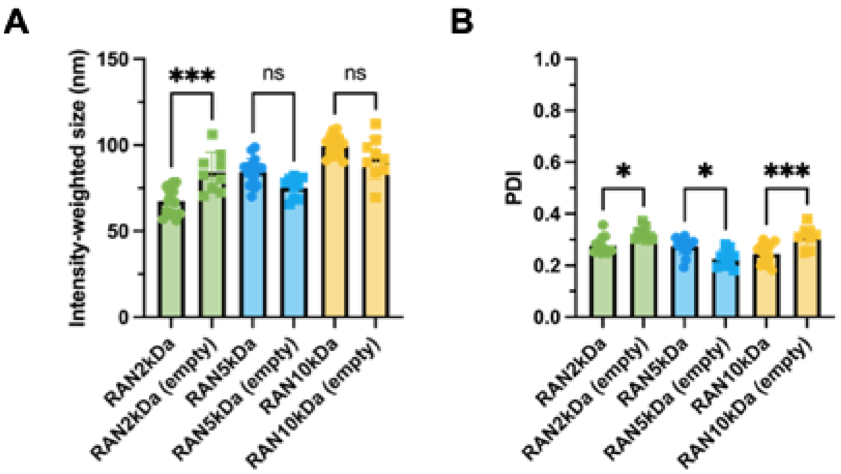

Figure S3. Physical characterization of empty RANs. Dynamic light scattering was used to measure (**A**) intensity-weighted size (diameter) and (**B**) PDI of RANs and empty RANs. *P* values determined by an ordinary one-way ANOVA test with Tukey's test for multiple comparisons. Replicates are experimental and technical, and data are shown as mean ± SD. ** signifies P<0.05, ** signifies P<0.01, *** signifies P<0.001*

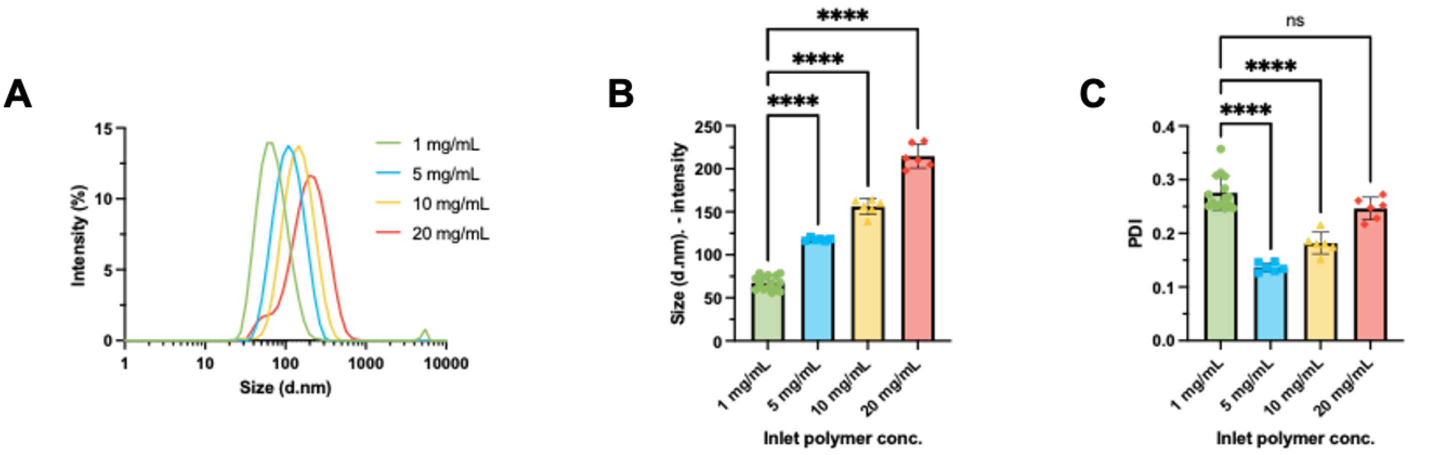

Figure S4. Polymer inlet concentration scale-up analysis. (**A**) Size distributions, (**B**) intensity-weighted size (diameter), and (**C**) PDI for empty RANs fabricated at increasing inlet polymer concentrations *(n=2-5 experimental replicates per group)*. *P* values determined by an ordinary one-way ANOVA test with Dunnett's test for multiple comparisons. Replicates are experimental and technical, and data are shown as mean ± SD. **** *signifies P<0.0001*

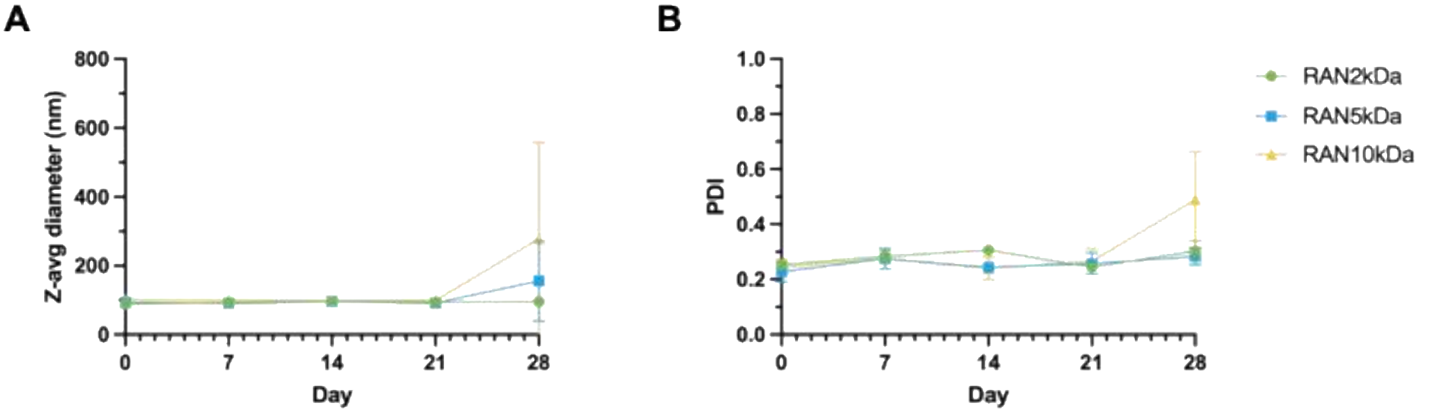

Figure S5. Long-term stability analysis. DLS measurements of (**A**) Z-average (intensity-weighted hydrodynamic size) and (**B**) polydispersity indices of RAN nanocarriers monitored over time *(n=2 per group)*. Replicates are biological, and data are shown as mean ± SD.

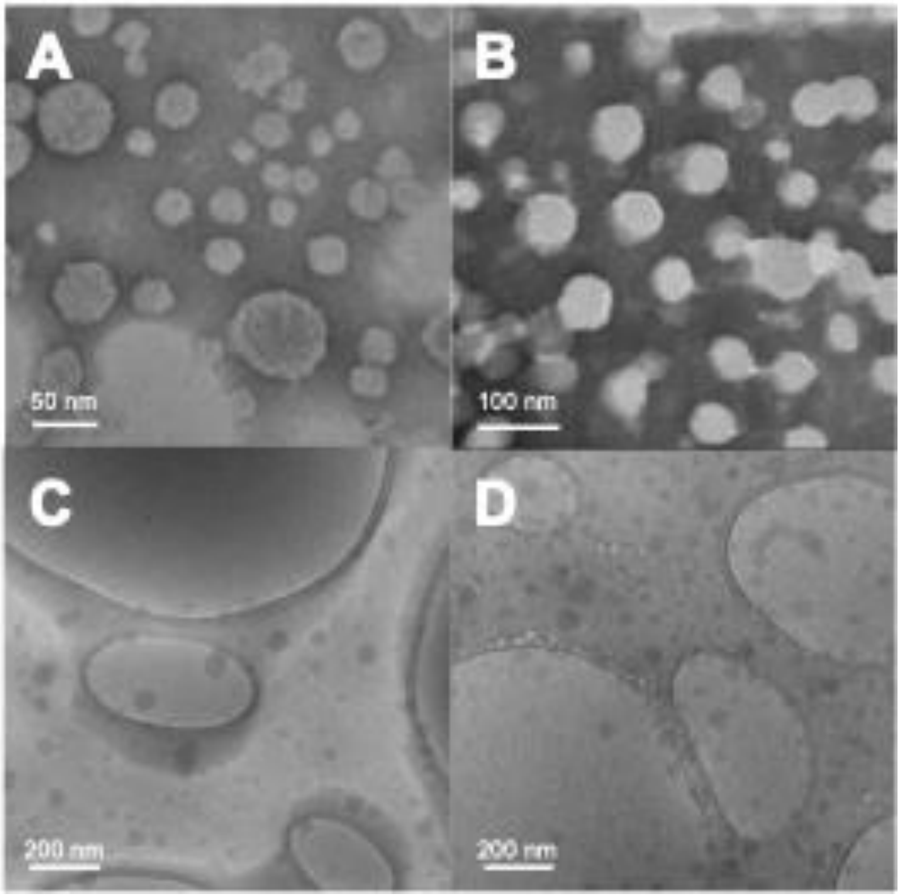

Figure S6. Transmission electron microscopy and cryogenic electron microscopy. Transmission electron microscopy (TEM) images of (**A**) RAN_{5kDa} and (**B**) RAN_{10kDa} nanocarriers. Cryogenic electron microscopy (cryoEM) images of (**C**) RAN_{5kDa} and (**D**) RAN_{10kDa} nanocarriers.

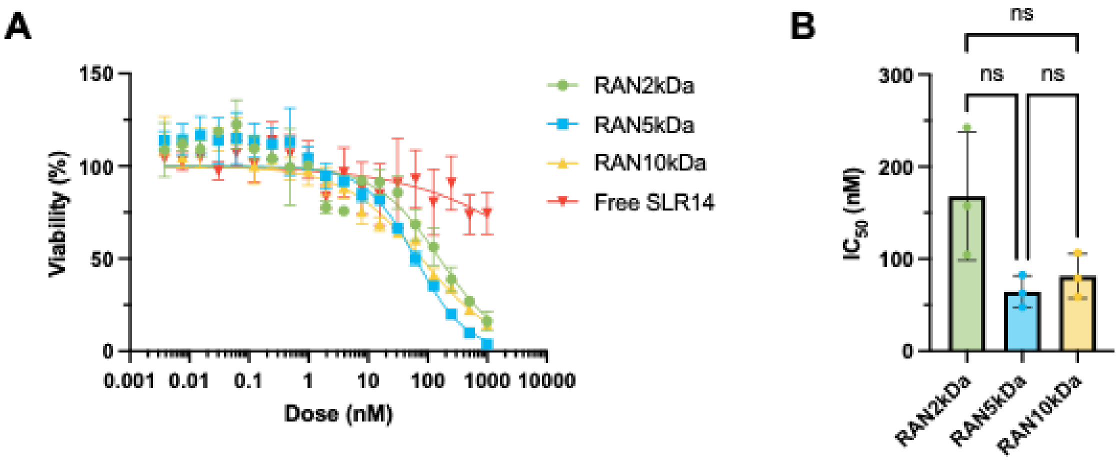

Figure S7. *In vitro* toxicity in A549-Dual cells. (**A**) Dose-dependent cytotoxicity sweep in A549-Dual reporter cells treated with RAN formulations and free SLR14 control *(n=2-3 per group)*, and (**B**) representative IC_{50} values. *P* values determined by an ordinary one-way ANOVA test with Dunnett's test for multiple comparisons. Replicates are biological, and data are shown as mean ± SD.

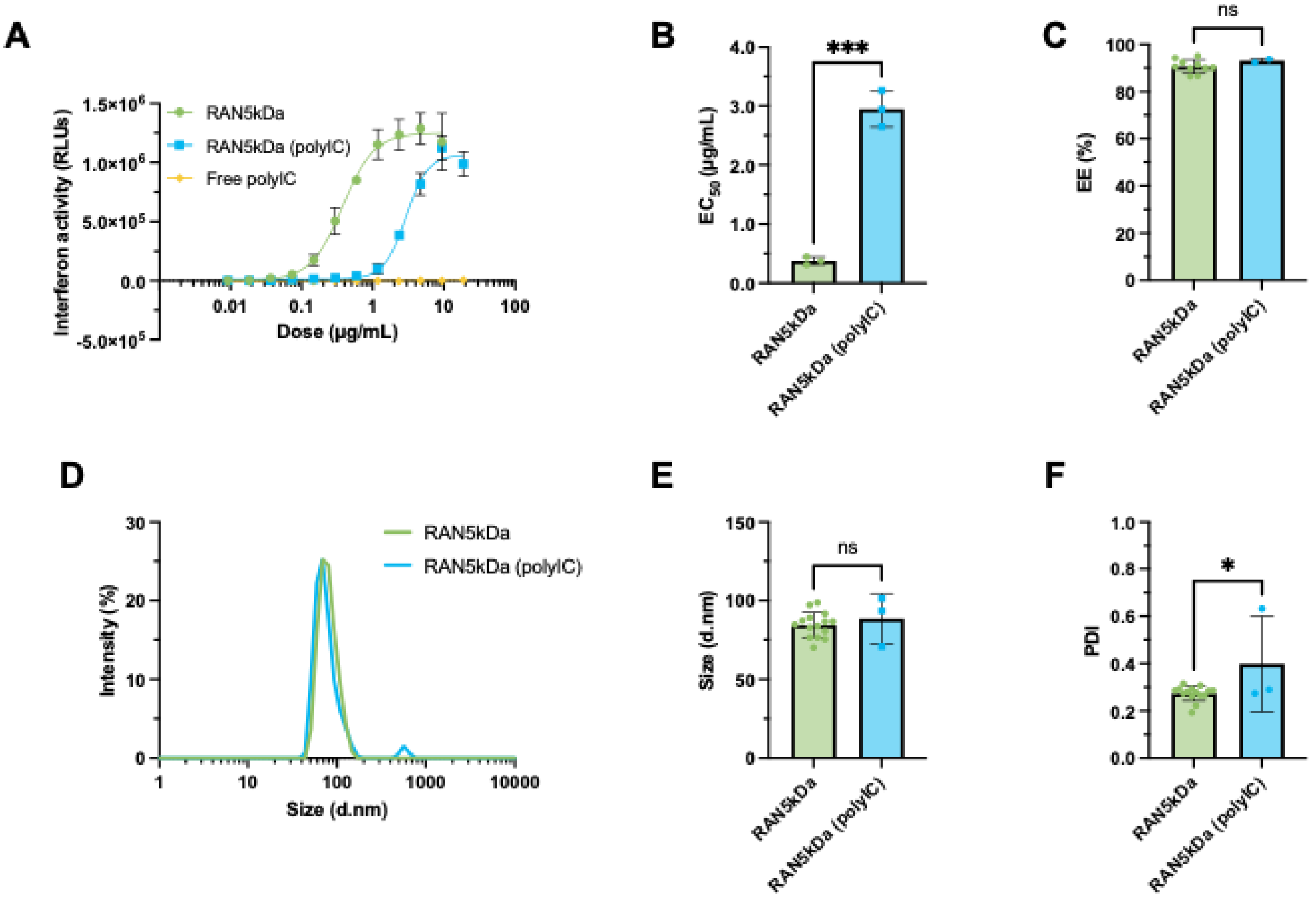

Figure S8. ***In vitro*** **activity and characterization of RANs loaded with polyIC.** (**A**, **B**) Upon treating A549-Dual reporter cells, RANs loaded with SLR14 induce an immune response at significantly lower doses than RANs loaded with polyIC. (**C**) polyIC was loaded into RANs at similar encapsulation efficiencies (EEs) as SLR14. (**D**, **E**, **F**) RANs loaded with polyIC possessed similar size and slightly higher polydispersity than RANs loaded with SLR14 *(n=3 per group)*. *P* values determined by an unpaired two-tailed t test. Replicates are biological, and data are shown as mean ± SD. ** signifies P<0.05*, **** signifies P<0.001*

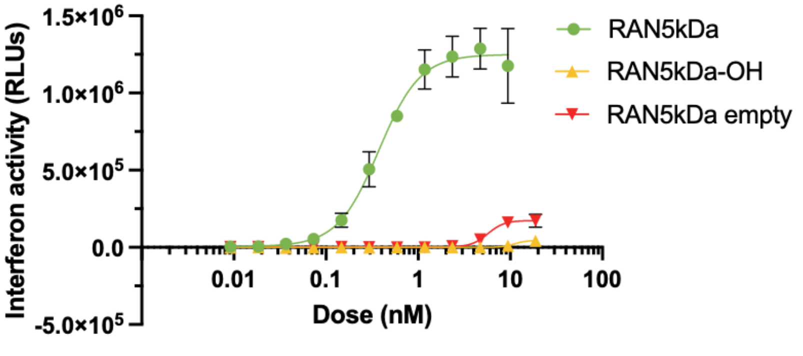

Figure S9. ***In vitro*** **activity with empty nanoparticle control.** Dose-dependent immunostimulatory activity in A549-Dual lung adenocarcinoma cells treated with RAN formulations and control groups including empty RANs and RANs loaded with an inactive SLR14-OH cargo *(n=3 per group).* Replicates are biological, and data are shown as mean ± SD.

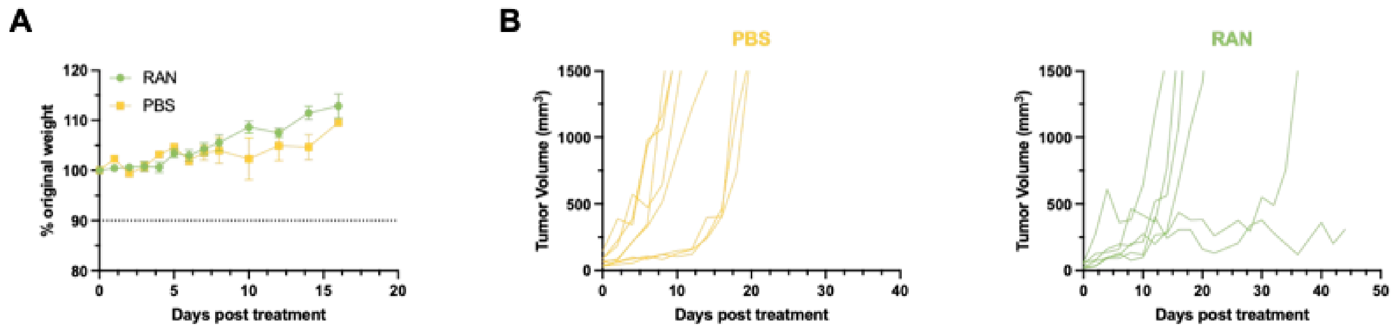

Figure S10. B16.F10 melanoma therapy study weight loss and spider plots. (**A**) Average weight of mice in the study measured throughout the treatment regimen, and (**B**) spider plots of individual mice tumor growth curves *(n=6-8 mice per group)*. Data are shown as mean ± SEM.

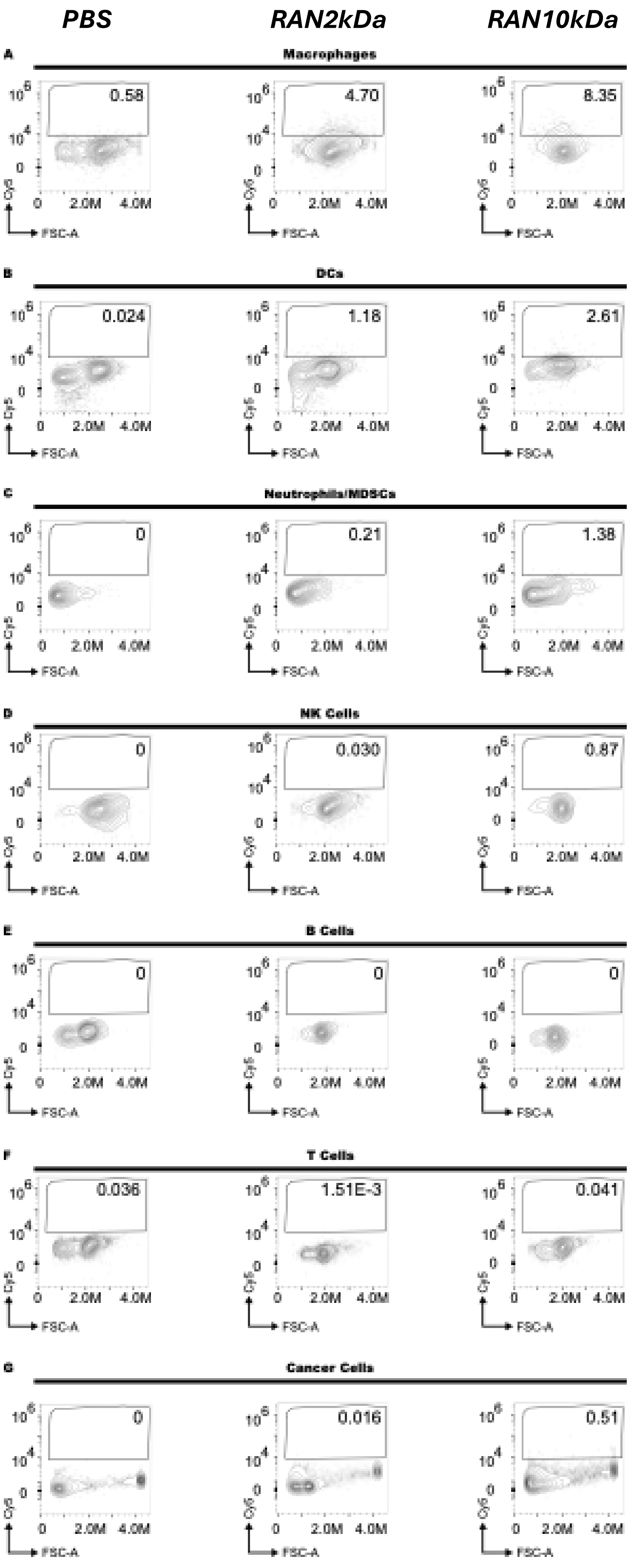

Figure S11. Flow cytometry gating schematic for analysis of Cy5-SLR14 uptake in the MC38 tumor microenvironment.
